# Diverse Trajectories Drive the Expression of a Giant Virus in the Oomycete Plant Pathogen *Phytophthora parasitica*

**DOI:** 10.3389/fmicb.2021.662762

**Published:** 2021-06-01

**Authors:** Sihem Hannat, Pierre Pontarotti, Philippe Colson, Marie-Line Kuhn, Eric Galiana, Bernard La Scola, Sarah Aherfi, Franck Panabières

**Affiliations:** ^1^Institut Hospitalo-Universitaire Méditerranée Infection, Marseille, France; ^2^MEPHI, Institut de Recherche pour le Développement, Aix-Marseille Université, Marseille, France; ^3^CNRS SNC5039, Marseille, France; ^4^Assistance Publique - Hôpitaux de Marseille, Marseille, France; ^5^INRAE, Université Côte d’Azur, CNRS, ISA, Sophia Antipolis, France

**Keywords:** gene transfer, giant viruses, *Phytophthora parasitica*, integration, endogenization, NCLDV, oomycetes

## Abstract

Giant viruses of amoebas, recently classified in the class Megaviricetes, are a group of viruses that can infect major eukaryotic lineages. We previously identified a set of giant virus sequences in the genome of *Phytophthora parasitica*, an oomycete and a devastating major plant pathogen. How viral insertions shape the structure and evolution of the invaded genomes is unclear, but it is known that the unprecedented functional potential of giant viruses is the result of an intense genetic interplay with their hosts. We previously identified a set of giant virus sequences in the genome of *P. parasitica*, an oomycete and a devastating major plant pathogen. Here, we show that viral pieces are found in a 550-kb locus and are organized in three main clusters. Viral sequences, namely RNA polymerases I and II and a major capsid protein, were identified, along with orphan sequences, as a hallmark of giant viruses insertions. Mining of public databases and phylogenetic reconstructions suggest an ancient association of oomycetes and giant viruses of amoeba, including faustoviruses, African swine fever virus (ASFV) and pandoraviruses, and that a single viral insertion occurred early in the evolutionary history of oomycetes prior to the *Phytophthora*–*Pythium* radiation, estimated at ∼80 million years ago. Functional annotation reveals that the viral insertions are located in a gene sparse region of the *Phytophthora* genome, characterized by a plethora of transposable elements (TEs), effectors and other genes potentially involved in virulence. Transcription of viral genes was investigated through analysis of RNA-Seq data and qPCR experiments. We show that most viral genes are not expressed, and that a variety of mechanisms, including deletions, TEs insertions and RNA interference may contribute to transcriptional repression. However, a gene coding a truncated copy of RNA polymerase II along a set of neighboring sequences have been shown to be expressed in a wide range of physiological conditions, including responses to stress. These results, which describe for the first time the endogenization of a giant virus in an oomycete, contribute to challenge our view of *Phytophthora* evolution.

## Introduction

Giant viruses of amoebas (GV), recently classified in the class Megaviricetes as part of the phylum Nucleocytoviricota, dramatically changed our view of the viral world following the description in 2003 of the first one, *Acanthamoeba polyphaga* mimivirus (La Scola et al., 2003). Since this initial discovery, dozens of GV have been isolated from the environment, animals, plants, and unicellular organisms (Delaroque and Boland, 2008; Derelle et al., 2008; Raoult and Boyer, 2010; Pagnier et al., 2013; Maumus et al., 2014; Sharma et al., 2014; Reteno et al., 2015; Leonard et al., 2018), or reconstructed from human and environmental metagenomes (Hingamp et al., 2013; Schulz et al., 2020). Accumulation of GV sequences has shown that these viruses display a mosaic genome architecture, encompassing a significant proportion of homologous sequences from viruses, eukaryotes, bacteria, and archaea, which were probably acquired by lateral gene transfers (Raoult et al., 2004; Filée et al., 2008). Taken together, these genes constitute a particularly original repertoire of hundreds of sequences, totally unprecedented in viruses (Colson et al., 2011a), which is still expanding (Moniruzzaman et al., 2020a; Rozenberg et al., 2020).

It is highly likely that invasion by GV deeply impacts the biology of their hosts. They may have deleterious effects upon integration within critical chromosomal regions. On the other hand, the extraordinary complexity of genes harbored by GV, such as transporters to take up nutrients, fermentation and photosynthesis genes, suggests that they may also complement some functions of their host (Vardi et al., 2012; Moniruzzaman et al., 2020a). Acquisition of novel functions provided by viral insertions may thus have dramatic consequences in the case of pathogenic microorganisms which have to cope with harsh and rapidly evolving environments. In this context, we identified a set of viral sequences possibly originating from a member of the Asfarviridae clade in the genome of the oomycete plant pathogen *Phytophthora parasitica* (Sharma et al., 2014).

Oomycetes constitute a deep lineage of lower eukaryotes that encompass some of the most notorious plant pathogens worldwide (Kamoun et al., 2015). Long time considered as fungi on the basis of structural features, shared ecological niches and common virulence strategies (Savory et al., 2015), oomycetes are grouped with brown algae diatoms and other unicellular organisms among Stramenopiles (Derelle et al., 2016). They include saprophytic organisms as well as highly aggressive animal and plant pathogens, such as the potato late blight agent or downy mildews. Most plant diseases are caused by members of the genus *Phytophthora*, which encompasses >180 formal species (Yang et al., 2017). The molecular bases of acquisition and evolution of virulence of these pathogens are a highly dynamic component that mainly relies on a sophisticated arsenal of effectors, most frequently extracellular proteins that are able to penetrate within host cells to defeat natural defense responses and manipulate plant functions to achieve successful infection (Franceschetti et al., 2017). Effectors may be secreted in the apoplast or in the cytoplasm, prior to reaching their site of action, which may be the cytoplasm, the plastid or even the nucleus.

In the present study, we aimed at gaining insights into the interactions between GV and the *Phytophthora* host genome by addressing the following questions: (i) Did the viral invasion occur one or several times during *Phytophthora*, and to a wider extent, oomycete evolution? (ii) Is it possible to trace the ancestral GV and approximately date the invasion event? (iii) What is the impact of GV insertions on the expression of the surrounding genomic environment?

## Materials and Methods

### Sequence Manipulation and Phylogenetic Analyses

The *P. parasitica* strain INRA-310 infected region was previously described on a single contig under the accession KI669605.1 (Sharma et al., 2014). This contig, designed contig 2.45, was used for subsequent searches. Predicted genes were ascribed to *Phytophthora* or GV genomes by best hit Blastp analyses against NR at GenBank, excluding *Phytophthora* sequences from the dataset, and by Blastp and tBlastn searches against the homemade database containing the GV genomes and their corresponding predicted proteins. Viral candidate sequences were retrieved in the oomycete genomes publicly available after tBlastn searches in the whole-genome contig subset of GenBank using default parameters. Conserved domains and putative functions of predicted proteins were searched using Blastp analyses on various databases, including PFAM (Mistry et al., 2020), Interpro (Blum et al., 2020), SMART (Letunic et al., 2020), and Superfamily (Pandurangan et al., 2019). Transposable elements (TEs) were searched by BlastN searches against the RepBase database using the Censor tool (Bao et al., 2015). A tRNA search was conducted using both tRNAscan-SE (Lowe and Chan, 2016) and Aragorn (Laslett and Canback, 2004). Sequences were aligned by using MUSCLE software (Edgar, 2004). Date of viral integration was estimated using the TimeTree tool (Kumar et al., 2017), based on different molecular clock methods (Matari and Blair, 2014). Phylogenetic trees were built by using the maximum likelihood method, which was carried out with the FastTree program (Price et al., 2010), with 1000 bootstrap replications, using JTT template substitution (standard default).

### *Phytophthora* Strains, Growth Media

*Phytophthora parasitica* strain INRA-310 was maintained in the INRAE ISA collection and grown on V8 medium at 24°C. *P. parasitica* mycelial cultures were submitted to diverse abiotic stresses as follows: initially, axenic cultures were grown at 37°C instead of 24°C to mimic heat shock. Alternatively, culture medium was supplemented with NaCl at a high concentration (0.6M), mimicking saline conditions encountered in drought. Finally, mycelial cultures were submitted to sub-lethal doses of CuSO_4_ (at a final 0.3M concentration). Initially employed as the famous Bordeaux mixture, this compound is commonly used in conventional agriculture to control oomycete diseases, at the increasing expense of environmental safety (Erwin and Ribeiro, 1996). Stress treatments were conducted for 4 h at 24°C. The mycelium was then harvested and thoroughly rinsed before nucleic acid extraction. Following these various treatments, expression of viral genes was assessed by quantitative RT-PCR.

### Expression Analyses

Expression of viral genes was assessed according two strategies. First, we estimated the total amount of reads matching each ORF of the contig in Blastn searches against RNA-Seq data available at GenBank (Accessions SRX1124837–SRX1124840, SRX1124842–SRX1124845, SRX1124847–SRX1124868, SRX27 27839–SRX2727852, SRX4902085–SRX4902107). In parallel, genes of interest were analyzed by qRT-PCR. For the RT-PCR, genomic DNA was extracted from 10 day-old cultures conducted on V8 medium as previously described (Panabières and Le Berre, 1999). RNA was extracted by using the RNeasy Mini Kit (QIAGEN, Germany). After the addition of RNAse, the DNA was digested by TURBO DNase (Invitrogen Thermo Fisher Scientific, Lithuania) at 37°C, three times in 30 min. Real-time RT-PCR was performed using two-step, one-step experiments.

For the two steps RT-PCR, cDNA was synthesized from 1 μg of RNA, using the SuperScript^TM^ VILO^TM^ cDNA Synthesis Kit Invitrogen (Thermo Fisher Scientific) reaction mixture following the manufacturer’s instructions. Gene expression was assessed by qPCR with gene-specific primers (Eurogentec, United States; Supplementary Table 1) and the fluorescent dye SYBR-Green (Invitrogen, Vilnius, Lithuania). The PCR amplification program consisted of: 95°C for 5 min following 45 cycles of 95°C for 10s, 59°C for 20s, and 72°C for 30s. Gene expression was considered efficient if there was an amplification curve with a Ct value ≤35 in each triplicate experiment with a melting temperature identical to those obtained on the DNA extracts (Table 3).

The one-step RT PCR reaction mixture consisted of RNA, using the SuperScript kit^TM^ III RT/Platinum^TM^ according to the manufacturer’s instructions. All assays were performed in triplicate and included negative controls (DNA/RNA-free PCR mix). A gene of *P. parasitica* strain INRA 310 encoding for the 40S ribosomal protein S3A (WS21, Genbank accession number: XM_008905737.1), known to be transcribed in all tested conditions, was used as an internal control (Yan and Liou, 2006).

### Sanger Sequencing

The PCR amplicon generated from purified cDNA of *P. parasitica* was sequenced on the Applied Biosystems 3130xl Genetic Analyzer (Thermo Fisher Scientific, France) using the BigDye Terminator DNA Sequencing Kit (Perkin-Elmer) according to the manufacturer’s instructions. The sequence was assembled using ChromasPro 2.0.0 software and compared to the NCBI database by Blastn analysis.

## Results

### Integration of GV Sequences in Oomycete Genomes

In the ∼550-kb contig from *P. parasitica* INRA-310 (contig 2.45, GenBank accession KI669605) 17 predicted genes matched with GV. When excluding the hits from *Phytophthora*, a total of 12 had GV hits as the best match (Sharma et al., 2014). This finding predated the achievement of several genome sequencing projects and the availability of an increasing number of complete genomes of oomycetes, including tens of *Phytophthora* genomes. Similarly, an invaluable number of studies on GV was conducted that totally modified our view of the viral world, expanding the phylogenetic tree of GV to thousands of members (Schulz et al., 2020). Therefore, we first performed a new gene calling step, prior to conducting sequential best Blast hit searches against public databases, excluding *P. parasitica*, then *Phytophthora*, and finally oomycetes from the datasets.

Gene calling revealed 7 additional ORFs of 180–1,005 nucleotides (60–335 codons) that were added to the ORFs already predicted in the contig, so that a total of 126 putative proteins were used as queries in the best Blast hit search, among which 15 appeared to better match GV sequences (Supplementary Table 2). These sequences mostly corresponded to partial protein sequences when compared to the best viral homologs, and notably encoded RNA polymerases I and II, the major capsid protein, and a DEAD helicase (Supplementary Table 2). We noted that the RNA polymerase II was represented by several ORFs, indicating a likely duplication or several transfer events (Supplementary Figure 1). The other viral sequences did not display any domain enabling functional annotation. No tRNA or ribosomal RNA genes were found across this contig.

Some viral candidate sequences were found to best match with oomycete sequences from *Phytophthora cactorum* or even *Pythium oligandrum*, whose genomes were released in public databases after the publication of the initial work (Armitage et al., 2018; Yang et al., 2018; Faure et al., 2020). This suggests a possible viral invasion of oomycete genomes having occurred before the *Phytophthora*-*Pythium* speciation. Alternatively, this might indicate that invasion events by GV occurred several times during oomycete evolution. Sequential best Blast hit searches did not reveal evidence of viral signatures that would be shared by all oomycetes. The viral candidates were therefore searched in oomycete genomes through tBlastn searches against the contig sequences hosted at the NCBI GenBank whole genome shotgun database. In line with independent reports (Yutin et al., 2014; Gallot-Lavallée and Blanc, 2017), we retrieved homologs of some of the viral candidate genes in several, but not all *P. parasitica* genomes publicly available and deposited under the denomination *P. parasitica* or the synonym *Phytophthora nicotianae* (Panabières et al., 2016). We also retrieved homologs in various *Phytophthora* species, as well as in at least three species formerly belonging to the *Pythium* genus, and recently ascribed to *Globisporangium*, among the order Pythiales (Uzuhashi et al., 2010), (Table 1). Therefore, the presence of GV sequences within the *P. parasitica* PPINRA-310 genome was not the outcome of a possible contamination, but rather reflected one or several viral insertion events affecting ancestral oomycetes.

**TABLE 1:** Distribution of the viral candidates from PPINRA-310 among oomycetes. Indicated are the species name and contig accession.

ORF number	Description	*Phytophthora*	Accession	*Pythium*	Accession
PPTG_14861	RNA Polymerase 2	*P. parasitica*		*Globisporangium irregulare*	NCVO01008180.1
		*P. quercina*	JACBOW010000075.1	*Globisporangium ultimum*	ADOS01001492.1
		*P. castanetorum*	JACBOV010000007.1	*Pythium oligandrum*	LSAJ01000108.1
		*P. ramorum*	RHLB01000784.1	*Globisporangium iwayamae*	AKYA02010070.1
		*P. boehmariae*	JAAVTJ010000040.1		
		*P. constricta*	JAAVTI010000058.1		
PPTG_14866	Major Capsid Protein	*P. parasitica*		*Globisporangium irregulare*	NCVO01008180.1
		*P. cactorum*	*NBIJ01015056.1*	*Globisporangium ultimum*	ADOS01001492.1
		*P. cryptogea*	*AUWJ02017648.1*		
		*P. constricta*	*JAAVTI010000058.1*		
		*P. quercina*	JACBOW010000075.1		
		*P. x alni*	*AUPN01065155.1*		
		*P. cambivora*	*AUVH01118500.1*		
PPTG_23628	T5orf172 domain protein	*P. parasitica*			
		*P. fragariae*	*QXGF01000047.1*		
PPTG_14881	RNA Polymerase 1	*P. parasitica*		*Globisporangium irregulare*	AKXZ02009300.1
		*P. cactorum*	*NBIJ01015056.1*		
		*P. syringae*	*JAAKBD010000413.1*		
		*P. vignae*	*JABJXB010000139.1*		
		*P. taxon totara*	*LGSO01000030.1*		
		*P. quercina*	JACBOW010000075.1		
		*P. x alni*	*AUPN01065155.1*		
		*P. cambivora*	*AUVH01118500.1*		
PPTG_14885	RNA Polymerase 1	*P. parasitica*		*Globisporangium irregulare*	NCVO01006854.1
		*P. rubi*	*QXFV01000589.1*		
		*P. cactorum*	*NBIJ01015056.1*		
		*P. x alni*	*AUPN01065155.1*		
		*P. cambivora*	*AUVH01118500.1*		
		*P. taxon totara*	*LGSO01000030.1*		
PPTG_14890	DEAD-like helicase	*P. parasitica*			
		*P. pinifolia*	*AWVW02037847.1*		
		*P. cactorum*	*NBIJ01015056.1*		
		*P. castanetorum*	JACBOV010000007.1		
		*P. ohioensis*	*JACBOX010000020.1*		
		*P. quercina*	JACBOW010000075.1		
		*P. pinifolia*	*AWVW02037847.1*		
		*P. cambivora*	*AUVH01118500.1*		
PPTG_14893	Hypothetical protein	*P. parasitica*		*Pythium periplocum*	MRVE01000072.1
		*P. cactorum*	*NBIJ01015056.1*	*Globisporangium ultimum*	AKYB02034954.1
				*Pythium oligandrum*	NAJK01000032.1
gene 787	Hypothetical protein	*P. parasitica*			
PPTG_14900	Hypothetical protein	*P. parasitica*			
gene 788	Hypothetical protein	*P. parasitica*			
gene 789	Hypothetical protein	*P. parasitica*			
PPTG_14924	Hypothetical protein	*P. parasitica*			
PPTG_14926	RNA Polymerase 2	*P. parasitica*		*Globisporangium irregulare*	NCVO01008180.1
				*Pythium oligandrum*	SPLM01000008.1
PPTG_14927	RNA Polymerase 2	*P. parasitica*		*Globisporangium irregulare*	NCVO01008180.1
				*Globisporangium ultimum*	ADOS01001492.1
		*P. ramorum*	RHLB01000784.1	*Pythium oligandrum*	LSAJ01000108.1
gene 404	Hypothetical protein	*P. parasitica*			
		*P. ohioensis*	JACBOX010000017.1		

The successive best Blast hit searches also revealed genes of ambiguous origin for which the 10 first hits corresponded to various unrelated organisms. Among them, we identified genes encoding a putative histone H3 (PPTG_14870), a DNA primase (PPTG_23627) and two partial ORFs (PPTG_14947 and PPTG_14951) corresponding to a ribonucleotide reductase. Such genes had already been predicted in several GV genomes (Derelle et al., 2008; Colson and Raoult, 2010; Yoshikawa et al., 2019). In addition, the histone gene was found to be restricted to *P. parasitica* and strikingly derived from canonical histones from oomycetes. Further phylogenetic analyses are necessary to precisely conclude whether this sequence is of viral origin or if it was acquired through lateral gene transfer involving other organisms.

### Invasion by an *Asfarviridae* Member Likely Occurred Once During Oomycete Evolution

**FIGURE 1:**
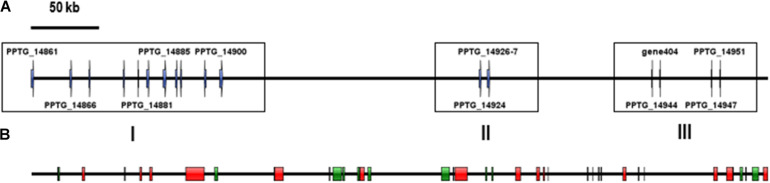
location of the viral candidate ORFs and identified TEs on the 550-kb contig. For clarity, *Phytophthora* predicted genes have not been integrated in the figure. **(A)** Viral candidates. Only ORFs with a known putative function are numbered. **(B)** Location of TE-derived domains identified using the Censor tool at Repbase. DNA transposons are represented by green blocks and retrotransposons are represented by red rectangles.

**FIGURE 2:**
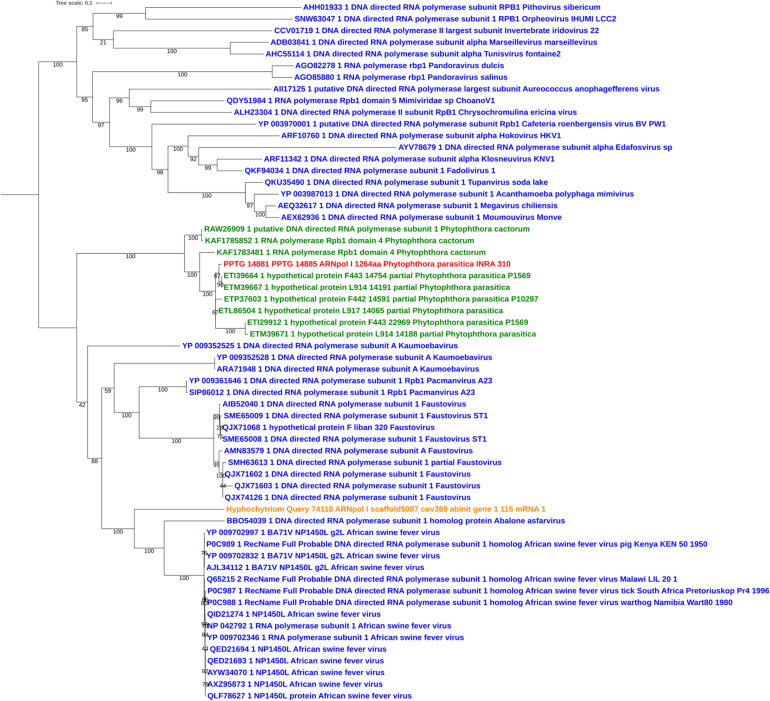
Maximum likelihood tree of RNA polymerase I amino acid sequences. The alignment was built using MUSCLE, and the iTol visualization was used with the rooted midpoint option. Sequences from NCLDVs are indicated in blue and sequences from oomycetes are indicated in green. The viral sequence from *P. parasitica* strain INRA-310 is indicted in red, and the viral sequence identified in the genome of *Hyphochytrium catenoides* (see text) is shown in orange. Bootstrap values are given below nodes in percent.

**FIGURE 3:**
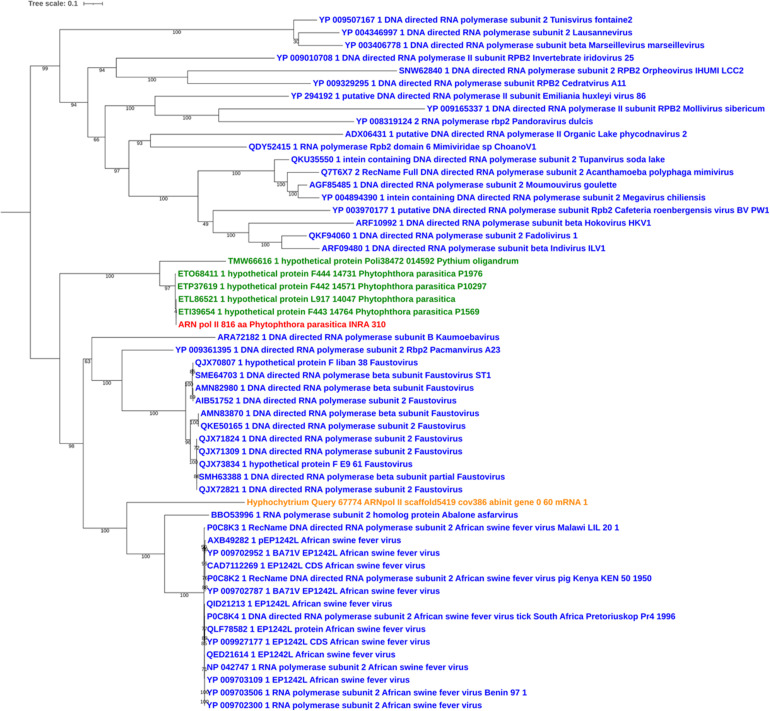
Maximum likelihood tree of RNA polymerase II protein. The alignment was built using MUSCLE, and the iTol visualization was used with the rooted midpoint option. Sequences from NCLDVs are indicated in blue and sequences from oomycetes are indicated in green. The viral sequence from *P. parasitica* strain INRA-310 is indicted in red, and the viral sequence identified in the genome of *Hyphochytrium* catenoides (see text) is shown in orange. Bootstrap values are given below nodes in percent.

Sequences of unambiguous viral origin were not located randomly across the contig 2.45, but rather accumulated at 3 discrete regions that spanned the whole contig length (Figure 1A). We thus observed that the two blocks encoding RNA polymerase II (PPTG_14861 and PPTG_14926/PPTG_14927p) were separated by >335 kb. Despite their overall conservation (78% identity, 85% positive over a 147-amino acids region, Supplementary Figure 1), these two genes might have originated from distinct viral donors and therefore from successive invasion events. In addition, looking at the best Blast hits indicated that the well-defined viral sequences found in *P. parasitica* (RNA Pol, Capsid, helicase), located in the blocks I and II, had better affinities to genes from asfarviruses, including Pacmanvirus, African swine fever virus (ASFV), and faustoviruses, while hypothetical proteins located at the block III better matched various viruses, with markedly less confident e-values. We then conducted a series of maximum likelihood phylogenetic analyses with a focus on RNA polymerases and the major capsid protein (MCP) to know whether the *P. parasitica* genome was invaded once by a single virus (or members of a single clade) or in successive events. Phylogenetic reconstructions showed that the viral sequences found in the genomes from oomycetes constitute a robust clade closely related to *Asfarviridae* (Figures 2, 3). Phylogenetic reconstruction based on the RNA polymerase II and the capsid protein suggested that the viral invasion occurred prior to the speciation between *Phytophthora* and *Pythium* (Figures 3 and Supplementary Figure 2). We included a sequence from RNA polymerase of viral origin found in the genome of *Hyphochytrium catenoides*, a basal organism belonging to Stramenopiles (Leonard et al., 2018). Several genes likely acquired from an *Asfarviridae* donor were identified within the genome of *H. catenoides*, among which were RNA polymerases and MCP (Gallot-Lavallée and Blanc, 2017; Leonard et al., 2018). The tree topologies indicate that *Hyphochytrium* more likely acquired viral genes from an asfarvirus (Figures 2, 3 and Supplementary Figure 2). The exact nature of the GV donor that invaded the *Phytophthora*/*Pythium* lineages is uncertain, but it clearly belonged to the group that includes asfarviruses and faustoviruses, which are relatives (Reteno et al., 2015). We could roughly estimate the date of integration of this donor into the genome of the *Phytophthora/Pythium* ancestor to 80 million years ago.

### Viral Sequences Are Imbedded Among Transposable Elements Within a Gene Sparse Region of the *P. parasitica* Genome

We intended to assign a function to genes present on the contig 2.45 in addition to likely viral sequences. Predicted proteins were generally of relatively small length (mean = 219, median = 166 amino acids). Identification of functional domains was achieved in only a few cases, so that the majority of ORFs encoded hypothetical proteins. Annotation revealed sequences relevant to protein-protein interactions, such as ankyrin repeat proteins, IPT/TIG domain proteins and chaperones, or components of the mRNA turnover and silencing. We also identified four members of the CRN (Crinkler and Necrosis) effector superfamily (Franceschetti et al., 2017), as well as several remnants of TEs (Supplementary Table 2). This observation prompted us to screen the whole contig for the presence of TEs and repetitive sequences. This task was difficult, because the *P. parasitica* strain INRA-310 genome was assembled from Illumina-derived data. Repetitive sequences including TEs are known to frequently escape identification in genomes. Indeed, their repetitive nature and their size, longer than the average read length generated by the Illumina technique, prevent their identification and lead to collapsing them in most genome assemblies (Panabières et al., 2020). Consequently, a substantial proportion of the contig length was made of N-stretches of unassembled regions, introducing numerous gaps in the assembly. Nonetheless, a search against RepBase revealed ∼40 TE-derived sequences that were scattered all along the contig 2.45, as already observed with viral sequences (Figure 1B). Further analysis revealed that retrotransposons were represented by 14 sequences, overwhelmingly corresponding to Gypsy-like elements, while class II transposons were mainly represented by Polintons, PiggyBac, MuDR, and Helitrons (Table 2). The paucity of protein-coding sequences associated to a wide abundance of TEs, which is itself highly underestimated because of the number of N-stretches in the contig, indicated that the viral insertion occurs in a gene sparse region.

**TABLE 2:** Distribution of Transposable elements across the contig 2.45.

Gene Id	Length (bp)	start	end
PiggyBac	642	18,925	19,567
PiggyBac	3,689	29,772	33,461
Copia	2,152	37,086	39,238
Polinton	494	68,331	68,825
Gypsy	1,509	79,718	81,227
Gypsy	2,082	86,958	89,040
Gypsy	13,700	113,775	127,475
MuDR	2,367	135,354	137,721
Polinton	164	179,461	179,625
Copia	6,247	179,840	186,087
Polinton	521	220,225	220,746
PiggyBac	6,299	223,052	229,351
Polinton	1,491	230,030	231,521
Polinton	1,720	240,788	242,508
Gypsy	3,298	242,878	246,176
Polinton	2,022	248,763	250,785
Polinton	6,016	303,155	309,171
PiggyBac	829	311,637	312,466
Gypsy	8,860	313,262	322,122
ISL2EU	908	335,963	336,871
Polinton	686	340,477	341,163
Gypsy	3,820	357,908	361,728
Gypsy	2,145	373,639	375,784
Gypsy	770	378,581	379,351
Polinton	268	382,048	382,316
Polinton	580	411,058	411,638
Helitron	248	415,117	415,365
Gypsy	506	419,285	419,791
MuDR	546	421,183	421,729
Gypsy	2,096	437,585	439,681
Mariner	483	448,659	449,142
Polinton	231	453,381	453,612
Gypsy	2,634	504,928	507,562
Gypsy	4,753	514,061	518,814
Polinton	397	518,992	519,389
Helitron	2,160	524,094	526,254
MuDR	556	528,211	528,767
Polinton	4,503	533,253	537,756
L1	2,985	541,466	544,451

**TABLE 3:** Results of SYBR Green RT-PCR performed on the cDNA *P. parasitica* INRA-310 prepared from mycelial cultures grown under different abiotic stress conditions. (P: Positive; N: Negative; Non-stressful condition: Control, stressful conditions with: 37°C, NaCl, CuSo4).

ORFs	Control	37°C	NaCl	CuSO_4_
PPTG_14900	P	N	N	P
PPTG_14924	P	N	P	P
PPTG_14926	P	N	N	P
PPTG_14884	P	N	P	N
PPTG_14866	P	N	N	P
PPTG_14927	P	N	P	P
Control (WS21)	P	P	P	P

### The Viral Locus Retained a Discrete Transcriptional Activity

To evaluate the potential impact of the viral sequences on *P. parasitica* biology, we intended to assess the expression of viral candidates in a variety of physiological situations. To this end, we first collected RNA-Seq data generated on *P. parasitica* that are publicly available. We mined libraries prepared from various pre-infection stages of *P. parasitica*, including libraries enriched in small RNAs. Overall, the contig was transcriptionally silent, although ∼20 ORFs organized into 10 short loci were expressed to various extent, as estimated by the total number of reads obtained in Blastn searches (Supplementary Table 3). Genes displaying some expression levels encoded CRNs, as well as proteases and hypothetical proteins, and a predicted tyrosine recombinase (PPTG_14945), possibly reflecting the activity of a TE. Sequences located in the block I were not expressed, while some ORFs from the blocks II and III were expressed to various extent. A refined analysis of RNA-Seq data from ORFs of the block I revealed that the RNA pol II-encoding gene PPTG_14861, as well as sequences corresponding to RNA pol I, were slightly expressed as small RNAs, which constitute potent non-coding RNA regulators (Supplementary Table 3). On the other hand, sequences from the block II encoding RNA pol II (represented by the partial sequences PPTG_14926 and PPTG_14927) were expressed in all situations observed. They were embedded among a set of genes that are also expressed in an apparent constitutive manner. Interestingly, this gene was located inside a set of genes that were also expressed. So, RNA pol II appeared to be the sole gene of viral origin being expressed under the conditions used for the generation of RNA-Seq data. We then investigated the potential expression of viral genes under various physiological conditions never explored to date. To this aim, qRT-PCR experiments by both SYBR Green and hydrolysis probe real-time methodologies were used.

Only six ORFs, namely PTG_14900, PPTG_14924, PPTG_14926, PPTG_14927, PPTG_14866, and PPTG_14884, appeared to be expressed using SYBR Green methodology in the control condition. Moreover, we assessed gene transcription under stress conditions consisting in a heat shock, NaCl and copper sulfate. As these stress experiments were known to mimic environmental conditions and to modify the level of expression of several *Phytophthora* genes, we aimed to assess these conditions may also affect the expression of the genes that have homologs with GV. Each of the six genes appeared to be expressed in at least one of the three stress conditions (Table 3). Notably, a positive result was found for PPTG_14927 in the 3 stress conditions, including heat shock, which usually leads to gene repression. Given the relatively low specificity of the SYBR green methodology, and intending to confirm these first results, we tentatively amplified these ORFs using specific hydrolysis probes. Interestingly, two of these six ORFs, PPTG_14926 and PPTG_14927, provided a significant expression signal in each situation, and displayed strongly reproducible results (Table 4). Further Sanger sequencing of the PCR products confirmed that the sequences amplified derived from the PPTG_14926 and PPTG_14927 targets. These experiments thus partially confirmed exploitation of RNA-Seq data, as no evidence of transcription was found for most genes, with the noticeable exception of PPTG_14926 and PPTG_14927, corresponding to a region of RNA polymerase II.

**TABLE 4:** Results of Platinum^TM^ RT-PCR performed on the RNA of *P. parasitica* INRA-310 prepared from mycelial cultures grown under different abiotic stress conditions. (N: Negative; Ct: Cycle threshold).

ORFs	Control			37°C			NaCl			CuSO_4_		
				
	Ct-1	Ct-2	Ct-3	Ct-1	Ct-2	Ct-3	Ct-1	Ct-2	Ct-3	Ct-1	Ct-2	Ct-3
PPTG_14900	N	N	N	N	N	N	N	N	N	N	N	N
PPTG_14924	N	N	N	N	N	N	N	N	N	N	N	N
PPTG_14926	28.0	28.0	28.1	29.2	29.4	28.7	29.7	29.9	30.0	29.9	29.9	29.8
PPTG_14884	N	N	N	N	N	N	N	N	N	N	N	N
PPTG_14866	N	N	N	N	N	N	N	N	N	N	N	N
PPTG_14927	29.6	29.5	29.8	26.4	27	26	27.2	27.3	26.7	26.2	25.6	25.2
Control (WS21)	24.8	25.1	27.3	21.8	20.7	21.4	23.8	25.6	23.2	24.1	24.0	29.2

## Discussion

The present study constitutes an additional step for the analysis conducted a few years ago that revealed the presence of GV sequences in the genome of the oomycete plant pathogen *P. parasitica*. Beyond this initial observation, we show here that viral insertion is a relatively ancient event, as it involved several *Phytophthora* species as well as members of *Globisporangium*, a new genus erected among the former *Pythium* genus (Uzuhashi et al., 2010). We first searched viral candidates among various *P. parasitica* genomes and confirmed their presence throughout the species, although to various extents. Such discontinuous distribution of viral sequences among *P. parasitica* isolates may have several explanations. First, viral insertions reside in a particularly dynamic region of the genome harboring TEs. Consequently, the majority of *P. parasitica* genomes that were sequenced according to the Illumina technique are poorly resolved at this locus, as illustrated by the substantial proportion of N-stretches found in the INRA-PP310 sequences or the small length of the contigs harboring viral sequences in the other genomes. Supporting this explanation is the finding that complete *P. parasitica* genomes sequenced by long read approaches, especially those of tobacco pathogens designated under the synonym *P. nicotianae* (Liu et al., 2016; Panabières et al., 2016), possess the viral insertions. Therefore, the lack of several ORFs, including the expressed region encoding RNA polymerase II, may result from differences in the sequencing methodology, and may also correspond to chromosomal deletions among strains. *P. parasitica* is considered to have a particularly broad host range, being able to infect more than 250 plant families (Panabières et al., 2016). And yet, frequent cases of host specificity are observed, and strains collected on tobacco have long been considered genetically distinct (Lacourt et al., 1994; Biasi et al., 2016). Specific searches relying on PCR-based identification conducted on a large range of *P. parasitica* strains, instead of Blast-based analyses conducted on a limited number of genomes available from *P. parasitica* strains, would be necessary prior to inferring relationships between the presence of viral insertions and the origin of the strains, with eventual consequences from these insertions on the virulence and host specificity of their recipient strains.

The discontinuous distribution of viral sequences that was observed among *Phytophthora* spp. is disconnected from the existing phylogeny of the genus *Phytophthora*, although *P. parasitica* shares taxonomical affinities among the phylogenetic clade 1 with *P. cactorum* (Yang et al., 2017) that appear to possess more conserved viral insertions than the other genomes analyzed. Finally, no trace of viral sequences was found outside *Phytophthora*, and in few *Pythium* members, when analyzing oomycetes. This observation is of interest, as the genus *Pythium* was for a long time considered a sister to the *Phytophthora* clade within a Pythiaceous lineage (Ascunce et al., 2017) before its recent redefinition into five genera (Uzuhashi et al., 2010). The conservation of viral sequences observed during the Blast searches therefore supports the hypothesis of a common ancestor being invaded by a single viral lineage shortly before the *Phytophthora*-*Pythium* radiation. In this evolutionary framework, the absence of any viral sequence in the known genomes of the *Hyaloperonospora* genus is worth noting. Indeed, this genus, represented by the obligate parasite *H. arabidopsidis*,appears closer to *Phytophthora* than *Pythium* in the majority of phylogenomic analyses (Matari and Blair, 2014; Ascunce et al., 2017; Mccarthy and Fitzpatrick, 2017). Similarly, there was no evidence of viral sequences in the complete genomes of downy mildews that have been clustered with *Phytophthora* spp. in these phylogenomic reconstructions. One possibility is that the lifestyle of these organisms, characterized by obligate parasitism, created a particular ecological niche which protected them from further viral invasions.

Phylogenetic analyses indicated that oomycete genomes were infected by several members of the family Asfarviridae, among which are Pacmanvirus, ASFV, and faustoviruses. This contrasts with findings by Moniruzzaman et al. (2020b), who identified viral sequences most similar to mimiviruses or phycodnaviruses in 12 algal genomes. The relatively modest genome sequence conservation among the asfarvirus relatives found here clearly shows that asfarvirus relatives, isolated and sequenced during the last decades (Reteno et al., 2015; Andreani et al., 2017), largely diverged from their common ancestor, or that new members of this clade have still to be uncovered. Asfarvirus giant relatives were mainly characterized following co-cultivation with amoebas or after isolation on natural hosts (Boyer et al., 2009; Pagnier et al., 2013). Recent surveys of metagenomic data have dramatically expanded the diversity of GV in terms of amounts of clades, but have not expanded the intra-clade diversity of Asfarviridae (Schulz et al., 2018, 2020; Bäckström et al., 2019). Therefore, we propose that the viral insertions observed in *P. parasitica*, and to a lesser extent in other oomycetes, reflect the sequences of an Asfarviridae ancestor rather than a member(s) of this clade that remains to be discovered. Oomycetes belong to Stramenopiles, which includes several organisms that have also integrated pieces of GV (Delaroque and Boland, 2008; Gallot-Lavallée and Blanc, 2017; Leonard et al., 2018). Recent phylogenetic studies showed that these various organisms retained sequences from a single different viral host. Notably, the deep branching *Hyphochytrium* and oomycetes display viral sequences of asfarviruses, whereas other stramenopiles were rather infected by a phaeovirus that infects marine algae (Gallot-Lavallée and Blanc, 2017). We note that infected oomycetes (members of *Phytophthora* and *Pythium, sensu lato*) and *Hyphochytrium* members are terrestrial, flagellated organisms, whereas the other analyzed stramenopiles are all marine microorganisms. This suggests that GV display some host specificity that is driven by ecological constraints. The six viral genes identified during this study have been frequently found in other cases of genome invasions and are shared by numerous GVs. They include the conserved genes encoding RNA polymerases I and II, the major capsid protein, and a helicase. This is puzzling and questions the significance and role of these genes. They were not eliminated, which may suggest that they are useful. Other sequences of likely viral origin without any evidence of a given function, the abundance of which is a hallmark of these GV genomes, have been characterized (Boyer et al., 2010; Schulz et al., 2017; Yoshikawa et al., 2019).

Furthermore, several genes harbored by the contig are of ambiguous origin and may be of viral origin as well. Among them, we found a histone, a DNA primase, a ribonucleotide reductase, and a plethora of hypothetical proteins which are restricted to *P. parasitica*. Whether they were acquired during viral infection is unknown, although their lateral acquisition is likely. On the other hand, the contig hosted few protein-coding sequences of oomycete origin, among them effectors, and is populated by a substantial proportion of TEs. This characteristic is found in oomycete and fungal genomes and is known as “two-speed genome” architecture, with regions enriched in TEs and pathogenicity-related genes (effectors) alternating with other gene-rich regions (Dong et al., 2015; Seidl and Thomma, 2017). Three sequences of probable oomycete origin were present in several copies across the contig, which are ankyrin repeat proteins, IPT/TIG domain proteins and CRN effectors. Ankyrins mediate protein-protein interactions, and thus participate in multiple cellular processes (Li et al., 2006). They also contribute to animal immunity and plant defense against pathogens (Ji et al., 2016; Ngaki et al., 2016). Conversely, ankyrins also act as effectors in the obligate bacterium *Anaplasma phagocytophilum* to manipulate host chromatin and gene expression (Rennoll-Bankert et al., 2015). Therefore, ankyrins might be a component of the *Phytophthora* virulence arsenal. Ankyrin repeat-containing proteins are also particularly prevalent in giant viruses (Iyer et al., 2006; Colson and Raoult, 2010; Mirzakhanyan and Gershon, 2020). One possibility is that the ankyrin-encoding ORFs uncovered on the contig 2.45 were acquired during viral invasion. Whether hybrid ankyrin complexes of both viral and cellular origins are established upon viral infection is unknown. Furthermore, the apparent lack of expression of the ankyrins present in the contig under the experimental conditions explored makes the function of these proteins obscure. Like ankyrins, IPT/TIG (immunoglobulin-like, plexins, transcription factors/transcription factor immunoglobulin) domains have been suggested to be involved in protein-protein interactions and DNA binding (Lara-Ramírez et al., 2017). Finally, CRN effectors constitute a large superfamily of proteins that have been identified across several kingdoms, but have been mainly studied in pathogenic oomycetes (Amaro et al., 2017). They are translocated into the host cells and contribute to virulence, and are thought to target the host nucleus and develop DNA-damaging activities (Stam et al., 2013; Camborde et al., 2019). Ankyrins, IPT/TIG proteins and CRNs may collectively fulfill related functions through targeting nuclear partners.

The contig analyzed in the present work also contained numerous repetitive sequences, among which are several known classes of TEs. This observation has important consequences. We may suppose that overall, TEs have been mobilized to interrupt the viral genes and protect the *Phytophthora* genome from the potential deleterious effects of viral invasion. Yet, the two main classes present in the contig, PiggyBac and Polintons, are frequently associated with viruses. PiggyBac was initially isolated from a baculovirus infecting the lepidopteran *Trichoplusia* (Gilbert et al., 2014), before further identification in a wide range of organisms, including oomycetes (Haas et al., 2009; Gaulin et al., 2018). This DNA transposon is also co-opted by herpesviruses, resulting in a new transposon entity that is able to infect fish genomes (Inoue et al., 2017). On the other hand, Polintons (aka Maveriks (Pritham et al., 2007) share several structural characteristics and an evolutionary ancestry with virophages, which are small (15–25 kb) dsDNA viruses that infect giant virus replication factories and may limit the replication of their viral host (La Scola et al., 2008; Mougari et al., 2019). Several analyses suggest that virophages are the progenitors of Polintons (Fischer and Suttle, 2011; Krupovic et al., 2014; Campbell et al., 2017). Polintons would then constitute a case of endogenization of viral elements. Therefore, the proportion of genes of viral origin in the contig 2.45 may be strikingly higher than initially proposed.

Using two different approaches, we show that at least one gene of viral origin is expressed in *P. parasitica* under a wide range of physiological conditions. Among the three stress conditions, only heat shock led to a decrease in genes. Hence, a copy of the RNA polymerase II is expressed at significant levels, as observed in qPCR experiments after analysis of RNA-Seq data. GV sequences have been previously identified in plant genomes, but they are transcriptionally silent (Maumus et al., 2014). However, traces of expression of viral sequences have been found in the transcriptome of the brown algae *Ectocarpus siliculosus* (Delaroque and Boland, 2008), another member of the Stramenopile lineage, like oomycetes. A close examination of expression data and of the architecture of the invaded contig reveals striking features. Hence, several distinct events may lead to inhibition of viral gene expression. First, interruption of the reading frames. This is clearly observed in the case of RNA Pol II (PPTG_14861), which only represents a truncated region of the viral homolog. We may suppose that the ancient, initial insertion of the viral gene was followed by successive mutations and rearrangements, likely driven by neighboring TEs. This agrees with the hypothesis of a massive mobilization of TEs that represents a large part of the contig, and which might have invaded it to inactivate the viral genes. Second, we observed that several viral genes are only transcribed in the form of small RNAs, although to a very moderate extent. It suggests that epigenetic mechanisms regulate their expression. There is compelling evidence that epigenetics, especially small RNA-based silencing, plays a major role in the biology of *Phytophthora*. Recent studies have shown that this mechanism targets protein coding genes like effectors and also TEs and other repetitive sequences like satellite DNA families (Vetukuri et al., 2012; Åsman et al., 2016; Panabières et al., 2020). Finally, viral genes may be silenced through fusion with TEs. Hence the gene encoding major capsid protein (PPTG_14866) is fused to a PiggyBac-derived sequence at its 3’end that likely contributes to its inactivation. In this context of global inactivation of viral insertions, the detection of the second RNA polymerase II gene using two non-redundant regions (PPTG_14926 and PPTG_14927) is enigmatic. Sequence alignment with the best viral hit revealed that the *P. parasitica* gene may correspond to a truncated copy that would be logically silent. In addition, we did not identify upstream sequences that would act as a potential promoter, but the 5’ moiety of the gene was flanked by a sequence corresponding to Harbinger, a DDE DNA transposon (Wicker et al., 2007). And it is located in a region containing 9 expressed ORFs, while the rest of the contig is globally transcriptionally silent. We may thus suppose a beneficial effect of this local environment, and that its transcription is driven by surrounding sequences, among which the TE is a good, but not the only, candidate. Although we showed that these two ORFs derived from the RNA polymerase II were transcribed, it is not certain that they were translated into functional proteins. However, their RNA might be used as a defense mechanism, as previously described for the moss *Physcomitrella patens*. Indeed, a recent analysis of the *P. patens* genome suggests that giant viruses embedded in the genome were transcribed during gametogenesis and were used as siRNA-mediated silencing to protect the gametes from viral infection (Lang et al., 2018). Whatever the transcriptional status of the RNA polymerase II in *P. parasitica*, it would indicate that viral insertion was followed by an endogenization process. Whether the transcription of this truncated RNA polymerase gene provides an advantage to *Phytophthora* is unclear, but undoubted, as it is the sole gene of viral origin to have escaped the inactivation process.

## Conclusion

This study constitutes the first description of a set of GV genes in the genome of an oomycete. The origins of these sequences largely remain obscure, but we here provide evidence that invasion of this class of major plant pathogens by a member of the family Asfarviridae was an ancient event. Our results raise intriguing questions about the relationship between TEs and viruses and possible shared steps in their evolutionary histories. These questions also pave the way for future studies aiming at a better knowledge of the nature and extent of the sequence flows that shaped *Phytophthora* genomes, with the evidence of lateral transfers. Also, traces of expression of at least two genes of viral origin indicates a likely GV endogenization event, and the general question of the importance of endogenous viral sequences in the evolution of eukaryotic genomes constitutes an active field of research (Geering et al., 2014; Frank and Feschotte, 2017; Moniruzzaman et al., 2020b). Our data adds to previous knowledge indicating that the presence of genes encoding RNA polymerase subunits and/or major capsid protein from GV in eukaryotic organisms appears to be a general phenomenon. Indeed, this was observed for various eukaryotes that are demonstrated or possible hosts of GV (Colson et al., 2011b; Maumus et al., 2014; Moniruzzaman et al., 2020b). A widespread endogenization of GV sequences was recently reported in various green algae, which consisted of large endogenous viral elements, and more fragmented footprints of past GV integration were also observed (Moniruzzaman et al., 2020b). These giant endogenous viral elements exhibited gene duplications and losses and were associated with introns invasions and transposons. The role and significance of viral sequences in the evolution of eukaryotic genomes continues to expand our knowledge of viral invasion and of the mosaicism of eukaryotic genomes. This is particularly important in the case of pathogenic microorganisms, as in the case of oomycetes, which are prone to permanent adaptation to their environment. An important contribution to this research would be the development of proteomics approaches to validate hypotheses arising from gene expression studies.

## Data Availability Statement

The datasets presented in this study can be found in online repositories. The names of the repository/repositories and accession number(s) can be found below: NCBI GenBank, accession no: NW_008634126.1, SRX1124837–SRX1124840, SRX1124842–SRX1124845, SRX1124847–SRX1124868, SRX27, 27839–SRX2727852, and SRX4902085–SRX4902107.

## Author Contributions

SA, BL, PP, and FP conceived the study. SH, M-LK, and EG conducted *Phytophthora* cultures and performed nucleic acids purification. FP and EG conducted manual annotation, data mining, and RNA-Seq analyses. SH and PP performed phylogenetic reconstructions. SH conducted qPCR analyses. SH, SA, and FP wrote the manuscript. BL and PC supervised the study. BL, PC, and EG revised the manuscript. All authors reviewed and approved the final manuscript.

## Conflict of Interest

The authors declare that the research was conducted in the absence of any commercial or financial relationships that could be construed as a potential conflict of interest.

## Supplementary Material

Supplementary Figure 1 Alignment of RNA Polymerase 2 encoding ORFs.

Supplementary Figure 2 Maximum likelihood tree of MCP. The figure legend is the same as in Figure 2.

Supplementary Table 1 primers used in this study.

Supplementary Table 2 Characteristics of predicted ORFs of the 550-kb contig from *P. parasitica* INRA-310 containing the ‘viral locus’. Sequences of likely viral origin are indicated in bold characters.

Supplementary Table 3 Relative expression of each ORF of the contig 2.45 as the total number of reads matching each sequence. *WS21* was used as internal constitutive control.
