# Supplementary material for: Diverse Trajectories Drive the Expression of a Giant Virus in the Oomycete Plant Pathogen Phytophthora parasitica
Source: Front Microbiol. 2021 Jun 1;12:662762. doi: 10.3389/fmicb.2021.662762 (PMC8204020; doi:10.3389/fmicb.2021.662762)
Supplement: Supplementary Figure 1 — Alignment of RNA Polymerase 2 encoding ORFs. [file Data_Sheet_1.PDF]

```
                   10        20        30        40        50        60
PPTG_14861 MTVLIKHNLHKVFVNGDWLGCVQDFGSFLDRYRHKRRVGEINMYTTVSHNIIANEIHMWV
           ::.. :::::::::::::::::.::. ::  ::.::: :::..:::::::: ::::::::
PPTG_14927 MTLVNKHNLHKVFVNGDWLGCVHDFALFLTIYRKKRRSGEIHLYTTVSHNIAANEIHMWV
         190       200       210       220       230       240

                   70        80        90       100       110       120
PPTG_14861 DSGRLIRPLLVVSNNMKDKDYTHEKFRQWINFKNDHVKQLRDGTIDIDDLAHEGIVEYIS
           :::::.::::::::::  : :::. :::::.. :::. ::.   :::::::..:::::::
PPTG_14927 DSGRLVRPLLVVSNNMAKKGYTHDTFRQWISLTNDHIAQLQARKIDIDDLARQGIVEYIS
         250       260       270       280       290       300

                  130       140
PPTG_14861 PEEHENTYIAFEHDRFKQHLKDPLHRY
           :::::::::::::::::::. :::::.
PPTG_14927 PEEHENTYIAFEHDRFKQHITDPLHRF
     310           320       330
```

Supplementary Figure S1
