# Supplementary figures and images for: Diverse Trajectories Drive the Expression of a Giant Virus in the Oomycete Plant Pathogen Phytophthora parasitica
Source: Front Microbiol. 2021 Jun 1;12:662762. doi: 10.3389/fmicb.2021.662762 (PMC8204020; doi:10.3389/fmicb.2021.662762)

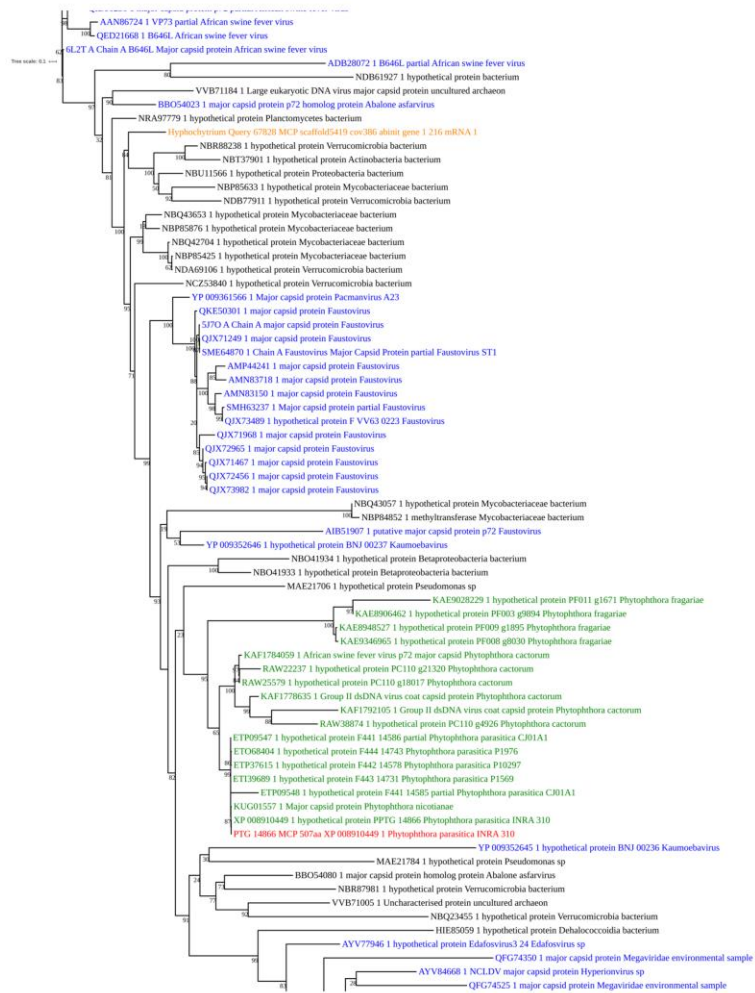

Supplementary Figure S2 (zoom)

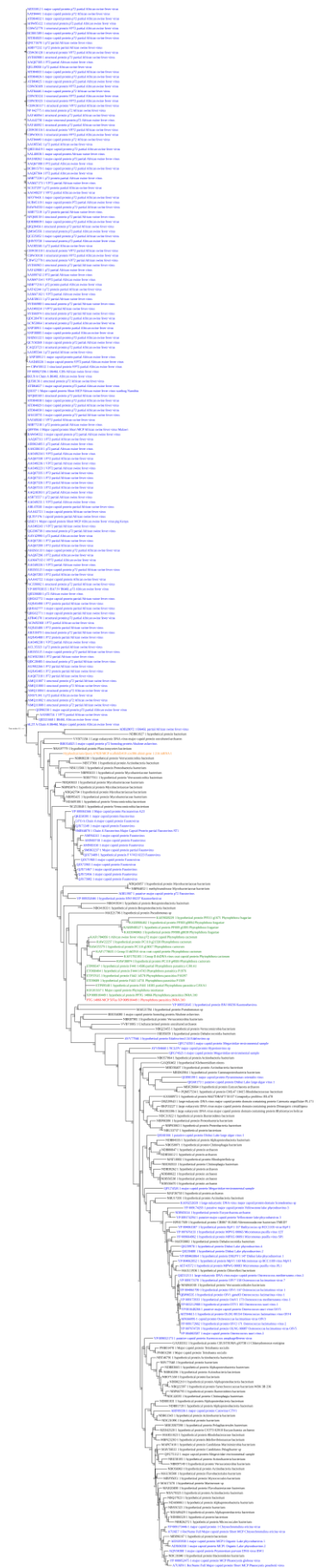

Supplementary Figure S2

Supplement: Supplementary Figure 2 — Maximum likelihood tree of MCP. The figure legend is the same as in Figure 2. [file Data_Sheet_2.PDF]
