# Supplementary material for: Diverse Trajectories Drive the Expression of a Giant Virus in the Oomycete Plant Pathogen Phytophthora parasitica
Source: Front Microbiol. 2021 Jun 1;12:662762. doi: 10.3389/fmicb.2021.662762 (PMC8204020; doi:10.3389/fmicb.2021.662762)
Supplement: Supplementary Table 1 — primers used in this study. [file Table_1.pdf]

Supplementary Table S1: Candidate-specific nucleotide sequences of primers and probes

Targeted organizations	sequences (5'-3')	Tm°C	Prob	Tm°C
		Tm°C		CG%
PPTG_10457 forward	CTGATACAGGAGCCAAGGTTG	54		
PPTG_10457 revers	AGGTCTGCATCTTCAAGAACA	50		
PPTG_14861 forward	GCCAGACCCTAGCAATACCT	54		
PPTG_14861 revers	TGTAAGCAGGCTCCTTGTGA	52		
PPTG_14900 forward	AAGGAGCCACATCACTTCGA	52	AGATGATGGCCCATTGCTCT	60
PPTG_14900 revers	CGATGTCGCACCTCTTAAGC	54		50
PPTG_14924 forward	ACGATGGGCCTCAACAAAAC	52	CCAGAAAGCTACGGACCGTA	62
PPTG_14924 revers	TCGCGTATCCATCGACAAGT	52		55
PPTG_14927 forward	CGATGCCGGTATGTCCTACT	55	GTCAATTTGCACGCGCTTTT	60
PPTG_14927 revers	GATCGCATTCATCAACGCCT	50		45
PPTG_14926 forword	ACATTATCAGCAAACCGGGC	52	GGCCGGGTTTAAGGAAATCG	62
PPTG_14926 revers	TTAGCGGAGTAGACCCCAAG	54		55
PPTG_16367 forward	CGGCTTGGACGGAAAACT	50		
PPTG_16367 revers	TGCTAGGGTTGACGTTATCCA	52		
gene_404 forward	CAAGCACAACTGTTAGCCGT	52		
gene_404f revers	TCGCTGATATGGCTACGGAT	52		
PPTG_14881 forward	CACCAATGCCACCACTGAAG	54		
PPTG_14881 revers	AGAGGTCCTTGGACACGATG	54		
PPTG_14885 forward	GGCACGGACATCAACATCAA	52		
PPTG_14885 revers	GTGATGGTTTGGATGGGGTG	54		
PPTG_14887 forward	ATCCGGAAATACAAGGGCCA	52		
PPTG_14887 revers	GATCTTGTCTTCCAACCGCC	54		
PPTG_14894 forward	TGGTCAAGAAGTGCAGCAAC	52		
PPTG_14894 revers	TCCGGCAATTTTCTCGCTTC	52		
PPTG_14893 forward	AAGCCAGTAAACCCCACGTA	52		
PPTG_14893 revers	CCAGCCCGTACGAATTCAAG	54		
PPTG_20267 forward	TCTCGCGAAGACCAAATCCT	52		
PPTG_20267 revers	CACGTCTCTGACCCTGAAGT	54		
PPTG_14884 forward	AACGTGAAGGAGCGGTTCTA	52	GATCTTCGTCCCCTCAGACC	64
PPTG_14884 revers	CCTCGGCGTCAAGATGAATG	54		60
PPTG_23628 forward	GATGATACATACCACGCGCG	54		
PPTG_23628 revers	TGCTGTTCTAGATCGCGGAT	52		

PPTG_14866 forward	TTTCTTCGCGGACATGGTTG	52	ACCCTGGTGAGCGTATCTTC	62
PPTG_14866 revers	AATGGGGTTCGAGTTGACCT	52		55
PPTG_14586 forward	GGATGTACGAAGAAACCGCG	54		
PPTG_14586 revers	TGTCTCCAGGTTGTTCGTGT	52		
gene_787 forward	GCTTTCGCCCATTGACTGTG	54		
gene_787 revers	CTAACAGTTCCTTAAGACGCCG	55		
gene_788 forward	CCTACTCGATGCGGTGTACA	54	ACCCAGTTGGAAGTTTTGCT	58
gene_788 revers	GTATCATGGATGGCTTGGCG	54		45
gene_789 forward	TAGCTGGCTGTCGAAAGTCA	52		
gene_789 revers	CACAGCGCAACTTCTTTCCA	52		
Control WS41 forward	TTCAAGTCCAGTGAGATCGG	52	CTGGAGGAGGAGGAGTAAGTA	64
Control WS41 revers	CGCATCACACAAAGACACAA	50		52
