# Supplementary material for: Diverse Trajectories Drive the Expression of a Giant Virus in the Oomycete Plant Pathogen Phytophthora parasitica
Source: Front Microbiol. 2021 Jun 1;12:662762. doi: 10.3389/fmicb.2021.662762 (PMC8204020; doi:10.3389/fmicb.2021.662762)
Supplement: Supplementary Table 2 — Characteristics of predicted ORFs of the 550-kb contig from P. parasitica INRA-310 containing the ‘viral locus’. Sequences of likely viral origin are indicated in bold characters. [file Table_2.pdf]

Supplementary Table S2: Characteristics of predicted ORFs of the 550-kb contig from *P. parasitica* PPINRA-310 containing the 'viral locus'.

ORF number	Length (aa)	Length (bp)	GC content	Description	*Excluding P. parasitica*	Query cover
**PPTG_14861**	**621**	**1867**	**44.77**	**RNA Polymerase 2**	***Pacmanvirus***	**98%**
PPTG_14862	436	1311	42.02	Hypothetical protein	*No hit*	
PPTG_14863	142	429	49.18	Hypothetical protein	*P. infestans*	75%
PPTG_14864	51	375	53.33	Hypothetical protein	*No hit*	
PPTG_14865	201	606	46.53	DNA J chaperone	*No hit*	
PPTG_23622	274	825	45.45	Hypothetical protein	*No hit*	
**PPTG_14866**	**507**	**1525**	**52.45**	**Major Capsid Protein**	***P. cactorum***	100%
PPTG_23623	914	2745	58.54	Transposase IS4	*P. megakarya*	100%
PPTG_23624	32	99	47.47	Hypothetical protein	*No hit*	
PPTG_23625	98	297	48.48	Hypothetical protein	*No hit*	
PPTG_23626	65	198	44.44	Hypothetical protein	*No hit*	
PPTG_14869	166	501	41.91	Hypothetical protein	*No hit*	
PPTG_23627	310	933	40.64	DNA primase	*Firmicutes bacterium*	46%
**PPTG_23628**	**297**	**892**	**41.36**	**T5orf172 domain protein**	***Wiseana iridescent virus***	**95%**
PPTG_14870	125	378	48.67	Histone H3	*Mus musculus*	90%
PPTG_14871	261	786	59.79	Hypothetical protein	*P. fragariae*	85
PPTG_14872	193	582	60.99	Hypothetical protein	*No hit*	
PPTG_14873	224	675	53.63	Ankyrin repeat protein	*Globisporangium splendens*	98%
PPTG_23629	85	258	53.48	Hypothetical protein	*No hit*	
PPTG_23630	307	924	53.99	Ankyrin repeat protein	*P. sojae*	21%
PPTG_14876	128	387	50.64	Hypothetical protein	*Nothophytophthora sp.*	98%
PPTG_23631	133	402	54.47	Ankyrin repeat protein	*P. fragariae*	62%
PPTG_14877	285	858	54.08	Argonaute	*P. fragariae*	47%
PPTG_14878	168	507	48.91	Argonaute	*P. megakarya*	86%
PPTG_14879	170	513	47.17	Hypothetical protein	*P. cactorum*	98%
PPTG_14880	130	393	56.48	Hypothetical protein	*P. cactorum*	100%
**PPTG_14881**	**507**	**1525**	**66.10**	**RNA Polymerase 1**	***P. cactorum***	96%
PPTG_14882	114	345	59.13	Early nodulin-75-like	*Lupinus angustifolius*	71%
PPTG_14883	160	483	53.00	Crinkler	*Aphanomyces invadans*	71%
PPTG_14884	286	861	51.91	RNAse H	*P. megakarya*	97%
**PPTG_14885**	**757**	**2275**	**62.33**	**RNA Polymerase 1**	***P. cactorum***	92%
PPTG_14886	176	531	53.10	Hypothetical protein	*Phytophthora cactorum*	86%

PPTG_14887	304	915	56.83	Rnase Y	*No hit*	
PPTG_14888	76	231	58.87	Hypothetical protein	*No hit*	
PPTG_23632	61	186	44.08	Hypothetical protein	*No hit*	
**PPTG_14890**	**414**	**1246**	**63,8**	**DEAD-like helicase**	***P. cactorum***	95%
PPTG_14891	191	576	56.07	PA14 domain	*P. cactorum*	97%
PPTG_14892	129	390	48.46	Hypothetical protein	*No hit*	
**PPTG_14893**	**286**	**862**	**46.28**	**Hypothetical protein**	***P. cactorum***	99%
PPTG_23633	110	333	48.34	Oxidoreductase/nitrogenase	*P. cactorum*	90%
PPTG_14894	678	2037	55.08	Ankyrin repeat protein	*Aphanomyces invadans*	31%
PPTG_14895	282	849	55.00	Hypothetical protein	*P. cactorum*	57%
PPTG_14896	200	603	62.02	Hypothetical protein	*P. cactorum*	100%
PPTG_14897	248	747	56.89	Hypothetical protein	*P. infestans*	75%
PPTG_23634	42	129	54.26	Hypothetical protein	*No hit*	
PPTG_14898	103	312	44.87	Hypothetical protein	*No hit*	
PPTG_14899	114	345	48.98	Hypothetical protein	*No hit*	
PPTG_23635	84	255	56.47	Hypothetical protein	*No hit*	
**gene 787**	**105**	**318**	**42.76**	**Hypothetical protein**	***Gaeavirus***	**81%**
**PPTG_14900**	**139**	**421**	**45.60**	**Hypothetical protein**	***Gaeavirus sp.***	**100%**
**gene 788**	**179**	**540**	**46.11**	**Hypothetical protein**	***Marseillevirus***	**87%**
**gene 789**	**188**	**567**	**47.79**	**Hypothetical protein**	***Paramecium bursaria chlorella virus***	**87%**
PPTG_14901	161	486	56.99	Hypothetical protein	*No hit*	
PPTG_23636	225	678	55.01	hAT family	*P. palmivora*	100%
PPTG_23637	85	258	57.36	Hypothetical protein	*P. megakarya*	51%
PPTG_14903	113	342	45.90	IPT/TIG domain-containing protein	*P. infestans*	83%
PPTG_14904	391	1176	54.15	Crinkler	*Aphanomyces invadans*	100%
PPTG_23638	541	1626	55.29	Periplasmic serine protease	*P. cactorum*	69%
PPTG_14907	83	704	54.97	Hypothetical protein	*No hit*	
PPTG_14909	161	486	52.05	DDE_Tnp_1-like zinc-ribbon	*P. rubi*	100%
PPTG_23639	324	975	53.43	Hypothetical protein	*P. palmivora*	98%
PPTG_23640	144	435	48.27	Hypothetical protein	*P. fragariae*	76%
PPTG_14912	288	867	46.94	Crinkler	*P. infestans*	99%
PPTG_23641	40	123	50.40	Hypothetical protein	*No hit*	
PPTG_23642	49	150	41.33	Hypothetical protein	*No hit*	
PPTG_23643	53	162	50.61	Hypothetical protein	*No hit*	
PPTG_14913	300	903	49.28	Tyrosine recombinase	*P. megakarya*	89%
PPTG_14917	196	591	54.82	Hypothetical protein	*P. megakarya*	99%
PPTG_23644	312	939	51.86	Hypothetical protein	*No hit*	

PPTG_14918	485	1458	51.51	retroviral pepsin-like	*P. fragariae*	86%
PPTG_14919	696	2091	53.51	Zinc knuckle	*P. rubi*	56%
PPTG_14920	569	1710	49.76	MULE transposase domain	*P. megakarya*	73%
PPTG_14921	668	2007	49.28	Peptidase_C48	*P. rubi*	100%
PPTG_14922	62	189	48.67	Hypothetical protein	*Phytophthora palmivora*	95%
PPTG_14923	104	315	55.87	Hypothetical protein	*No hit*	
PPTG_23645	74	225	52.86	Hypothetical protein	*No hit*	
**PPTG_14924**	**393**	**1182**	**48.77**	**Hypothetical protein**	***Pyramimonas orientalis virus***	**47%**
PPTG_23646	103	312	48.71	Integrating conjugative element protein	*No hit*	
PPTG_14925	353	1062	51.22	Hypothetical protein	*P. rubi*	100%
**PPTG_14926**	**119**	**172**	**46.51**	**RNA Polymerase 2**	***Pythium oligandrum***	84%
**PPTG_14927**	**384**	**1156**	**52.07**	**RNA Polymerase 2**	***Pythium oligandrum***	54%
PPTG_14928	215	647	49.84	IPT/TIG domain-containing protein	*P. cactorum*	97%
PPTG_14929	178	667	46.32	IPT/TIG domain-containing protein	*P. cactorum*	98%
PPTG_14931	353	1062	51.79	pro-apoptotic serine protease	*P. infestans*	73%
PPTG_23647	116	351	49.57	Hypothetical protein	*P. sojae*	88%
PPTG_14932	95	288	50.34	Hypothetical protein	*P. sojae*	85%
PPTG_14933	166	501	47.70	Cadherin domain	*P. infestans*	84%
PPTG_14934	62	189	55.55	Hypothetical protein	*Aphanomyces stellatus*	100%
PPTG_23648	95	288	59.37	Hypothetical protein	*No hit*	
PPTG_23649	102	309	44.33	Hypothetical protein	*No hit*	
PPTG_23650	77	234	43.16	Hypothetical protein	*P. cactorum*	66%
PPTG_23651	73	222	50.90	Pro-apoptotic serine protease	*P. megakarya*	100%
PPTG_23652	101	306	59.47	Pro-apoptotic serine protease	*P. cactorum*	76%
PPTG_14935	462	1389	55.94	Hypothetical protein	*P. infestans*	43%
PPTG_14936	171	615	54.47	Hypothetical protein	*No hit*	
PPTG_14937	272	895	43.24	Hypothetical protein	*Aphanomyces invadans*	95%
PPTG_23653	168	507	49.70	Hypothetical protein	*Notophytophthora*	83%
PPTG_14938	137	414	53.38	Hypothetical protein	*No hit*	
PPTG_23654	336	1011	53.01	Tc5 transposase	*P. megakarya*	48%
PPTG_23655	185	558	49.41	Hypothetical protein	*P. megakarya*	75%
PPTG_14941	201	606	53.79	Hypothetical protein	*P. rubi*	84%
PPTG_14942	119	360	50.27	Crinkler	*P. fragariae*	100%
PPTG_14943	130	393	58.01	pleiotropic drug resistance transporter	*P. fragariae*	96%
PPTG_14944	148	447	62.86	Ankyrin repeat protein	*Pythium oligandrum*	30%
PPTG_14945	1018	3057	55.67	Tyrosine recombinase	*P. infestans*	67%
gene 403	59	180	45.55	Hypothetical protein	*No hit*	

gene 404	174	525	39.61	Hypothetical protein	*Abalone asfarvirus*	86%
PPTG_23656	89	270	41.48	Hypothetical protein	*No hit*	
gene 407	187	564	35.46	Polyprotein	*P. palmivora*	98%
PPTG_14946	443	1332	49.77	IPT/TIG domain-containing protein	*P. cactorum*	83%
PPTG_14947	78	237	42.61	Ribonucleotide reductase	*Pythium oligandrum*	83%
PPTG_23657	488	1467	55.21	Hypothetical protein	*P. fragariae*	56%
PPTG_14950	178	537	55.86	Hypothetical protein	*P. megakarya*	83%
PPTG_14951	123	372	43.01	Ribonucleotide reductase	*Pythium oligandrum*	100%
PPTG_23658	541	1626	49.87	Pre-mRNA splicing Prp18-interacting factor	*P. cactorum*	43%
PPTG_23659	68	207	59.42	Zinc knuckle	*P. infestans*	92%
PPTG_14954	286	861	54.24	Hypothetical protein	*No hit*	
PPTG_14955	110	333	53.15	Hypothetical protein	*P. cactorum*	94%
PPTG_14956	253	762	53.41	SH3 domain protein	*P. cactorum*	95%
PPTG_14957	108	327	44.03	Hypothetical protein	*P. megakarya*	98%
PPTG_23660	104	315	45.07	Hypothetical protein	*Notophytophthora sp. Chili*	77%
PPTG_23661	71	216	41.66	Hypothetical protein	*No hit*	
PPTG_14958	238	717	49.79	RNAse H - copia	*P. sojae*	63%
PPTG_14959	129	390	44.10	Hypothetical protein	*P. rubi*	100%
PPTG_14960	190	573	55.49	Endonuclease-reverse transcriptase	*P. infestans*	96%
PPTG_23662	557	1674	54.30	Hypothetical protein	*P. infestans*	98%
gene 757	335	1008	40.57	Hypothetical protein	*Bacteroidetes bacterium*	78%

e-value	PerIdent	Accession	*Excluding Phytophthora*	Query cover	e-value	PerIdent (%)	Accession
**3,00E-141**	**40.03**	**YP_009361395.1**					
4,00E-53	75.70	XP_002904539.1	*No hit*				
0.0	68.37	**RAW25579.1**	***Pacmanvirus***	**97%**	**3,00E-119**	**37.09%**	**YP_009361566.1**
0.0	58.38	OWZ18198.1	*Aphanomyces astaci*	64%	2,00E-116	35.93%	XP_009829632.1
5,00E-07	27.61	PKM60256.1					
**1,00E-17**	**30.00**	**YP_004732953.1**					
4,00E-46	65.49	NP_001074488.1					
3,00E-72	52.82	KAE9302549.1	*Globisporangium splendens*	75%	6,00E-12	32.04%	KAF1318305.1
1,00E-77	58.69	KAF1313111.1					
2,00E-14	62.50	XP_009528308.1	*Pythium brassicum*	21%	7,00E-15	58.46%	TYZ69092.1
9,00E-41	55.71	RLN67412.1					
1,00E-06	36.90	KAE8882535.1	*Aphanomyces invadans*	82%	4,00E-05	32.43%	XP_008861487.1
7,00E-32	49.64	KAE9355519.1	*Nothophytophthora*	47%	3,00E-24	43.79%	RLN10159.1
8,00E-59	66.21	OWZ23573.1	*Nothophytophthora*	86%	2,00E-42	51.03%	RLN10159.1
2,00E-80	71.86	RAW30228.1	*Nothophytophthora*	100%	2,00E-76	70.59%	RLN51472.1
3,00E-33	48.12	KAF1783482.1	*Nostocales cyanobacterium*	73%	6,00E-07	35.00%	NTW21441.1
0.0	75.48	**KAF1783481.1**	***African swine fever virus***	**92%**	**9,00E-79**	**34.17%**	**AYW34070.1**
7,00E-05	35.87	XP_019418554.1					
1,00E-34	54.39	RHY18145.1					
4,00E-110	58.39	OWY97948.1	*Globisporangium splendens*	70%	4,00E-73	53.78%	KAF1314281.1
0.0	67.81	**RAW26909.1**	*Globisporangium splendens*	73%	0.0	63.58%	**KAF1318322.1**
3,00E-08	31.06	RAW36420.1	*No hit*				

6,00E-158	57.25	**KAF1785851.1**	***African swine fever virus***	**94%**	**2,00E-45**	**29.50%**	**QID21238.1**
2,00E-53	58.58	KAF1786218.1	*Plasmopara halstedii*	29%	9,00E-05	50.88%	XP_024574916.1
8,00E-115	61.62	**KAF1786224.1**	***Faustovirus***	**52%**	**0.002**	**24.71%**	**AMN83493.1**
3,00E-12	40.78	KAF1786224.1	*No hit*				
2,00E-22	35.21	RHY18145.1					
1,00E-72	71.60	RAW28203.1	*Nothophytophthora*	82%	2,00E-12	27.08%	RLN81096.1
3,00E-38	47.37	RAW21218.1	*No hit*				
1,00E-67	58.06	XP_002898459.1	*Nothophytophthora*	63%	2,00E-19	39.24%	RLN97743.1
**8,00E-06**	**31.87%**	**AYV79931.1**					
**9,00E-20**	**38.89**	**AYV79931.1**					
**2,00E-11**	**28.48%**	**QBK86218.1**					
**1,00E-11**	**29.94%**	**ABT13981.1**					
8,00E-88	61.11	POM57552.1	*Nothophytophthora*	100%	1,00E-49	41.04%	RLN87599.1
1,00E-17	81.82	OWZ08808.1	*Nothophytophthora*	55%	6,00E-14	68.09%	RLN92296.1
3,00E-43	75.53	KAF4131735.1	*Plasmopara halstedii*	83%	1,00E-39	70.21%	XP_024582831.1
5,00E-96	45.73	RHY22287.1					
0.0	81.05	RAW35315.1	*Peronospora effusa*	69%	0.0	75.07%	RMX64955.1
1,00E-67	58.79	KAE9282245.1	*Nothophytophthora*	87%	1,00E-28	45.95%	RLN87515.1
3,00E-117	51.04	POM69866.1	*Nothophytophthora*	95%	1,00E-46	33.12%	RLN86594.1
8,00E-15	45.76	KAE8933464.1	*Plasmopara halstedii*	73%	5,00E-14	35.29%	XP_024576663.1
0.0	91.99	XP_002899381.1	*Lagenidium giganteum*	99%	2,00E-140	69.31%	AHF51834.1
2,00E-130	63.20	OWY99381.1	*Aphanomyces euteiches*	96%	3,00E-105	52.76%	KAF0731635.1
3,00E-58	57.44	OWZ14121.1	*Nothophytophthora*	86%	2,00E-17	37.50%	RLN87380.1

3,00E-115	47.22	KAE8962597.1	*Pythium brassicum*	50%	5,00E-13	27.72%	TYZ60733.1
1,00E-175	62.17	KAE9280906.1	*Nothophytophthora*	20%	4,00E-06	24.66%	RLN50778.1
0.0	58.81	OWY97904.1	*Nothophytophthora*	60%	1,00E-75	36.39%	RLN53846.1
0.0	46.26	KAE9285904.1	*Globisporangium splendens*	93%	1,00E-69	29.64%	KAF1328639.1
6,00E-15	62.71	POM64112.1	*No hit*				
**6,00E-08**	**27.23**	**QOI90536.1**					
6,00E-148	58.36	KAE9035112.1	*Aphanomyces euteiches*	92%	2,00E-112	49.85%	KAF0723802.1
4,00E-48	78.43	TMW66616.1	***Pacmanvirus***	**73%**	**6,00E-19**	**42.05%**	**YP_009361395.1**
2,00E-80	64.60	TMW66616.1	***Pacmanvirus***	**79%**	**2,00E-68**	**42.17%**	**YP_009361395.1**
1,00E-112	84.21	RAW39954.1	*Plasmopara halstedii*	93%	4,00E-97	75.62%	XP_024582831.1
3,00E-100	82.29	KAF1783978.1	*Plasmopara halstedii*	96%	2,00E-65	62.21%	XP_024582831.1
2,00E-149	79.39	XP_002902521.1	*Peronospora effusa*	73%	1,00E-139	75.34%	RMX64955.1
2,00E-38	60.19	XP_009518215.1	*Nothophytophthora*	88%	7,00E-18	40.78%	RLN91235.1
1,00E-26	64.20	XP_009520510.1	*Pythium insidiosum*	83%	2,00E-10	40.51%	GAX98881.1
8,00E-77	86.52	KAF4040823.1	*Plasmopara halstedii*	96%	4,00E-48	53.42%	XP_024580271.1
2,00E-31	96.77	KAF0685871.1					
2,00E-13	52.44	KAF1786217.1	*Pythium oligandrum*	55%	4,00E-09	45.95%	TMW56834.1
6,00E-29	93.15	OWZ24340.1	*Globisporangium splendens*	100%	2,00E-28	91.78%	KAF1328045.1
4,00E-35	76.84	RAW35315.1	*Nothophytophthora*	56%	7,00E-25	91.23%	RLN58634.1
4,00E-127	89.95	XP_002902334.1	*No hit*				
3,00E-96	57.92	RHY22287.1					
3,00E-09	28.17	RLN94206.1					
1,00E-76	71.17	OWZ21105.1	*Pythium insidiosum*	48%	5,00E-48	55.21%	GAY03384.1
1,00E-39	52.14	OWY98050.1	*Globisporangium splendens*	69%	2,00E-19	42.74%	KAF1330881.1
1,00E-51	57.31	KAE9050197.1	*Aphanomyces stellatus*	88%	2,00E-45	50.00%	KAF0713297.1
7,00E-50	69.77	KAE8928552.1	*Aphanomyces stellatus*	97%	6,00E-48	67.77%	KAF0713297.1
4,00E-50	69.05	KAE8920983.1	*Nothophytophthora*	96%	7,00E-49	68.25%	RLN87471.1
2,00E-15	77.78	TMW59009.1					
4,00E-158	58.28	KAF4136654.1	*Nothophytophthora*	23%	4,00E-57	54.50%	RLN27402.1

3,00E-20	36.84%	BBO54025.1					
3,00E-71	60.33%	POM68737.1	*uncultured archaeon*	89%	1,00E-04	26.16%	VVB71255.1
0.0	82.11	RAW39954.1	*Nothophytophthora*	89%	7,00E-136	60.28%	RLN49513.1
2,00E-18	66.15	TMW56829.1					
5,00E-93	54.68	54.68% KAE9063084.1	*No hit*				
3,00E-25	38.46	OWY95643.1	*No hit*				
2,00E-54	69.11	TMW56829.1					
2,00E-130	74.05	RAW35315.1	*Peronospora effusa*	47%	7,00E-116	65.27%	RQM14192.1
3,00E-12	49.21	KAF4040382.1	*No hit*				
2,00E-30	51.92	KAF1784847.1	*No hit*				
6,00E-101	64.26	*RAW35305.1*	*Plasmopara halstedii*	95%	9,00E-89	56.98%	XP_024577550.1
7,00E-31	53.77	OWY95349.1	*Nothophytophthora*	97%	1,00E-19	39.05%	RLN89317.1
6,00E-22	49.38	RLN60421.1					
1,00E-53	57.65	XP_009531753.1	*Plasmopara halstedii*	63%	2,00E-44	48.24%	XP_024580567.1
8,00E-67	83.72	KAE9007988.1	*Plasmopara halstedii*	100%	1,00E-51	65.89%	XP_024580566.1
1,00E-99	83.51	KAF4138707.1	*Nothophytophthora*	95%	2,00E-77	63.59%	RLN70632.1
0.0	80.27	KAF4148747.1	*Plasmopara halstedii*	98%	0.0	70.38%	XP_024581962.1
3,00E-10	26.32%	NBP65151.1					

Excluding Oomycetes	Query cover	e-value	PerIdent (%)	Accession	Repbase output
Stylophora pistillata	72%	7,00E-37	24.96%	XP_022788383.1	PiggyBac
No hit					
Batrachochytrium salamandrivorans	98%	6,00E-71	50.83%	OON06960.1	
No hit					
No hit					
Hydra vulgaris	59%	0.002	30.38%	XP_004211345.1	
No hit					
Gigaspora margarita	98%	6,00E-17	35.16%	KAF0343232.1	
Phytophthora water mold MELD virus	98%	2,00E-71	71.51%	DAC81615.1	
					hAT
No hit					
No hit					
Pacmanvirus	**92%**	**3,00E-122**	**32.58%**	**YP_009361646.1**	

No hit					
No hit					Gypsy
No hit					Gypsy
No hit					
Rozella allomycis	100%	2,00E-18	29.64%	RKP18223.1	
Octopus bimaculoides	48%	1,00E-06	53.66%	XP_014786661.1	
Ectocarpus siliculosus	66%	8,00E-07	37.33%	CBJ25816.1	
Batrachochytrium dendrobatidis	57%	6,00E-23	30.80%	XP_006680738.1	
Proteobacteria bacterium	52%	1,00E-76	46.36%	PZN31256.1	
No hit					PiggyBac
No hit					
No hit					
[Klebsormidium nitens	92%	2,00E-34	32.72%	GAQ92861.1	
					Gypsy
Guillardia theta	99%	4,00E-60	37.99%	XP_005830398.1	
No hit					PiggyBac

No hit					Gypsy
No hit					Gypsy
Plasmodiophora brassicae	55%	8,00E-23	27.93%	SPQ96400.1	MULE
No hit					MULE
Gracilariopsis chorda	52%	6,00E-38	40.32%	PXF40870.1	Harbinger
Thalassiosira pseudonana	94%	3,00E-31	38.10%	XP_002290869.1	
Ectocarpus siliculosus	84%	4,00E-15	37.67%	CBJ25816.1	
Planoprotostelium fungivorum	74%	4,00E-42	34.77%	PRP82453.1	
No hit					
No hit					
No hit					
Campylobacter jejuni	100%	2,00E-21	70.97%	TKH92731.1	Gypsy
					Gypsy
Batrachochytrium dendrobatidis	100%	1,00E-21	71.23%	XP_006677811.1	
Rhizophagus irregularis	69%	6,00E-15	52.11%	PKC54878.1	
					Gypsy
Rozella allomycis	64%	1,00E-24	35.56%	EPZ34517.1	
No hit					
Thalictrum thalictroides	38%	2,00E-34	52.63%	KAF5177759.1	Pogo
Serendipita vermifera	85%	4,00E-10	31.06%	PVG03481.1	
No hit					
No hit					
Ectocarpus siliculosus	74%	5,00E-07	33.67%	CBN79954.1	
Cavenderia fasciculata	31%	1,00E-11	65.91%	XP_004355180.1	
No hit					

Aureococcus anophagefferens	47%	1,00E-16	33.48%	XP_009039296.1	
Fistulifera solaris	92%	1,00E-15	48.61%	GAX13727.1	
					Gypsy
					Gypsy
Flavobacteriales bacterium	88%	3,00E-30	47.71%	RYF09989.1	
bacterium MnTg04	44%	3,00E-44	34.77%	GBF30817.1	
					Gypsy
No hit					
Brachionus plicatilis	73%	2,00E-07	34.18%	RMZ99520.1	
No hit					
Phytophthora water mold MELD virus	46%	6,00E-13	34.23%	DAC81621.1	Polinton
No hit					Polinton
Daphnia pulex	66%	2,00E-19	35.66%	EFX61361.1	L1
No hit					
