# Supplementary material for: Diverse Trajectories Drive the Expression of a Giant Virus in the Oomycete Plant Pathogen Phytophthora parasitica
Source: Front Microbiol. 2021 Jun 1;12:662762. doi: 10.3389/fmicb.2021.662762 (PMC8204020; doi:10.3389/fmicb.2021.662762)
Supplement: Supplementary Table 3 — Relative expression of each ORF of the contig 2.45 as the total number of reads matching each sequence. WS21 was used as internal constitutive control. [file Table_3.pdf]

Supplementary Table S3: Relative expression of each ORF of the contig 2.45 as the total number of reads matching each sequence. *WS21* was used as internal constitutive control.

| SRA accession | Library | Total reads | *WS21* | PPTG_14861 | PPTG_14862 | PPTG_14863 | PPTG_14864 | PPTG_14865 | PPTG_23622 | PPTG_14866 | PPTG_23623 | PPTG_23624 | PPTG_23625 |
|---|---|---|---|---|---|---|---|---|---|---|---|---|---|
| SRX4902085 | zoospores | 32,586,376 | 1297 | 0 | 0 | 0 | 0 | 0 | 0 | 0 | 0 | 0 | 0 |
| SRX4902087 | zoospores | 31,690,213 | 1694 | 0 | 0 | 0 | 0 | 0 | 0 | 0 | 1 | 0 | 0 |
| SRX4902088 | zoospores | 31,640,390 | 1561 | 0 | 0 | 0 | 0 | 0 | 0 | 0 | 0 | 0 | 0 |
| SRX4902094 | zoospores | 31,868,614 | 1702 | 0 | 0 | 0 | 0 | 0 | 0 | 0 | 0 | 0 | 0 |
| SRX4902093 | 3 h germinated cysts | 33,566,333 | 2653 | 0 | 0 | 0 | 0 | 0 | 0 | 0 | 1 | 0 | 0 |
| SRX4902105 | 3 h germinated cysts | 29,209,817 | 2550 | 0 | 0 | 0 | 0 | 0 | 0 | 0 | 2 | 0 | 0 |
| SRX4902106 | 3 h germinated cysts | 34,411,485 | 2427 | 0 | 0 | 0 | 0 | 0 | 0 | 0 | 2 | 0 | 0 |
| SRX4902107 | 3 h germinated cysts | 32,286,422 | 2271 | 0 | 0 | 0 | 0 | 0 | 0 | 0 | 1 | 0 | 0 |
| SRX4902095 | 12 h sporulating hyphae | 33,067,780 | 1545 | 2 | 0 | 0 | 0 | 0 | 0 | 0 | 2 | 0 | 0 |
| SRX4902096 | 12 h sporulating hyphae | 33,388,830 | 1268 | 0 | 0 | 0 | 0 | 0 | 0 | 0 | 0 | 0 | 0 |
| SRX4902098 | 12 h sporulating hyphae | 33,381,053 | 1219 | 0 | 0 | 0 | 0 | 0 | 0 | 0 | 1 | 0 | 0 |
| SRX4902097 | 24 h sporulating hyphae | 32,357,379 | 1542 | 0 | 0 | 0 | 0 | 0 | 0 | 0 | 0 | 0 | 0 |
| SRX4902103 | 24 h sporulating hyphae | 31,945,743 | 1379 | 0 | 0 | 0 | 0 | 0 | 0 | 0 | 0 | 0 | 0 |
| SRX4902104 | 24 h sporulating hyphae | 31,344,661 | 978 | 0 | 0 | 0 | 0 | 0 | 0 | 0 | 1 | 0 | 0 |
| SRX4902086 | 6 day sporulating hyphae | 35,459,486 | 753 | 0 | 1 | 0 | 0 | 0 | 0 | 0 | 1 | 0 | 0 |
| SRX4902089 | 6 day sporulating hyphae | 33,587,404 | 985 | 0 | 0 | 0 | 0 | 0 | 0 | 0 | 0 | 0 | 0 |
| SRX4902091 | 6 day sporulating hyphae | 32,030,660 | 788 | 0 | 0 | 0 | 0 | 0 | 0 | 0 | 0 | 0 | 0 |
| SRX4902092 | 6 day sporulating hyphae | 32,474,405 | 948 | 0 | 1 | 0 | 1 | 0 | 0 | 0 | 0 | 0 | 0 |
| SRX4902100 | vegetative hyphae | 32,030,660 | 931 | 0 | 0 | 0 | 0 | 0 | 0 | 0 | 0 | 0 | 0 |
| SRX4902101 | vegetative hyphae | 32,474,405 | 1203 | 1 | 0 | 0 | 0 | 0 | 0 | 0 | 0 | 0 | 0 |
| SRX4902102 | vegetative hyphae | 35,459,486 | 1087 | 0 | 0 | 0 | 0 | 0 | 0 | 1 | 2 | 0 | 0 |
| SRX2727852 | Mycelium RNA | 41,871,236 | 2004 | 0 | 0 | 0 | 0 | 0 | 0 | 0 | 0 | 0 | 0 |
| SRX2727851 | Mycelium RNA | 53,641,916 | 6918 | 0 | 0 | 0 | 0 | 0 | 0 | 0 | 2 | 0 | 0 |
| SRX2727850 | Mycelium RNA | 41,575,074 | 2747 | 0 | 0 | 0 | 0 | 0 | 0 | 0 | 0 | 0 | 0 |
| SRX2727845 | Mycelium small RNA | 11,755,441 | 4 | 11 | 12 | 0 | 3 | 5 | 3 | 1 | 33 | 0 | 0 |
| SRX2727844 | Mycelium small RNA | 19,332,985 | 8 | 18 | 48 | 4 | 3 | 20 | 4 | 4 | 74 | 0 | 2 |
| SRX2727843 | Mycelium small RNA | 13,745,664 | 2 | 20 | 41 | 1 | 4 | 15 | 10 | 4 | 60 | 0 | 2 |

| PPTG_23626 | PPTG_14869 | PPTG_23627 | PPTG_23628 | PPTG_14870 | PPTG_14871 | PPTG_14872 | PPTG_14873 | PPTG_23629 | PPTG_23630 | PPTG_14876 | PPTG_23631 | PPTG_14877 | PPTG_14878 | PPTG_14879 |
|---|---|---|---|---|---|---|---|---|---|---|---|---|---|---|
| 0 | 0 | 0 | 0 | 0 | 0 | 0 | 0 | 10 | 0 | 0 | 0 | 0 | 0 | 0 |
| 0 | 0 | 0 | 0 | 0 | 0 | 0 | 0 | 11 | 0 | 0 | 0 | 0 | 0 | 0 |
| 0 | 0 | 1 | 0 | 0 | 0 | 0 | 0 | 14 | 0 | 0 | 0 | 0 | 0 | 0 |
| 0 | 0 | 0 | 0 | 0 | 0 | 0 | 0 | 9 | 0 | 0 | 0 | 0 | 0 | 0 |
| 0 | 0 | 0 | 0 | 0 | 0 | 0 | 0 | 25 | 0 | 0 | 0 | 0 | 0 | 0 |
| 0 | 0 | 0 | 0 | 0 | 0 | 0 | 0 | 21 | 0 | 0 | 0 | 0 | 0 | 0 |
| 0 | 0 | 0 | 0 | 0 | 0 | 0 | 0 | 20 | 0 | 0 | 0 | 0 | 0 | 0 |
| 0 | 0 | 0 | 0 | 0 | 0 | 0 | 0 | 12 | 0 | 0 | 0 | 0 | 0 | 0 |
| 0 | 0 | 0 | 0 | 0 | 0 | 2 | 0 | 23 | 0 | 0 | 0 | 0 | 0 | 0 |
| 0 | 0 | 1 | 0 | 0 | 0 | 0 | 0 | 21 | 0 | 0 | 0 | 0 | 0 | 0 |
| 1 | 0 | 0 | 0 | 0 | 0 | 0 | 0 | 28 | 1 | 0 | 0 | 0 | 1 | 0 |
| 0 | 0 | 0 | 0 | 0 | 0 | 0 | 0 | 27 | 0 | 0 | 0 | 0 | 0 | 0 |
| 0 | 1 | 1 | 0 | 0 | 1 | 0 | 0 | 22 | 0 | 0 | 0 | 0 | 0 | 0 |
| 0 | 0 | 0 | 0 | 0 | 0 | 0 | 0 | 13 | 0 | 0 | 0 | 0 | 0 | 0 |
| 0 | 0 | 0 | 0 | 0 | 0 | 0 | 0 | 9 | 0 | 0 | 0 | 0 | 0 | 0 |
| 0 | 0 | 0 | 0 | 0 | 0 | 0 | 0 | 32 | 0 | 0 | 0 | 0 | 0 | 0 |
| 0 | 0 | 0 | 0 | 0 | 0 | 0 | 0 | 20 | 0 | 0 | 0 | 0 | 0 | 0 |
| 0 | 0 | 0 | 0 | 0 | 0 | 0 | 0 | 11 | 0 | 0 | 0 | 0 | 0 | 0 |
| 0 | 0 | 0 | 0 | 0 | 0 | 0 | 0 | 25 | 0 | 0 | 0 | 0 | 0 | 0 |
| 0 | 0 | 0 | 0 | 0 | 0 | 0 | 0 | 23 | 0 | 0 | 0 | 0 | 0 | 0 |
| 0 | 0 | 0 | 0 | 0 | 0 | 0 | 0 | 39 | 0 | 0 | 0 | 0 | 0 | 0 |
| 0 | 0 | 0 | 0 | 0 | 0 | 0 | 0 | 7 | 0 | 0 | 0 | 0 | 0 | 0 |
| 0 | 0 | 0 | 0 | 0 | 0 | 0 | 0 | 31 | 0 | 0 | 0 | 0 | 0 | 0 |
| 0 | 0 | 0 | 0 | 0 | 0 | 0 | 0 | 22 | 0 | 0 | 0 | 0 | 0 | 0 |
| 0 | 0 | 2 | 3 | 0 | 5 | 2 | 3 | 3 | 1 | 4 | 0 | 0 | 0 | 0 |
| 0 | 0 | 0 | 3 | 1 | 6 | 4 | 8 | 2 | 4 | 19 | 3 | 0 | 1 | 0 |
| 2 | 4 | 6 | 3 | 2 | 2 | 2 | 8 | 3 | 1 | 9 | 2 | 0 | 0 | 0 |

| PPTG_14880 | PPTG_14881 | PPTG_14882 | PPTG_14883 | PPTG_14884 | PPTG_14885 | PPTG_14886 | PPTG_14887 | PPTG_14888 | PPTG_23632 | PPTG_14890 | PPTG_14891 | PPTG_14892 | PPTG_14893 | PPTG_23633 |
|---|---|---|---|---|---|---|---|---|---|---|---|---|---|---|
| 0 | 0 | 0 | 0 | 0 | 0 | 0 | 0 | 0 | 0 | 0 | 0 | 0 | 0 | 0 |
| 0 | 0 | 0 | 0 | 0 | 0 | 0 | 0 | 0 | 0 | 0 | 0 | 0 | 0 | 0 |
| 0 | 0 | 0 | 0 | 0 | 0 | 0 | 0 | 0 | 0 | 0 | 0 | 0 | 0 | 0 |
| 0 | 0 | 0 | 0 | 0 | 0 | 0 | 0 | 0 | 0 | 0 | 0 | 0 | 0 | 0 |
| 0 | 0 | 0 | 0 | 0 | 0 | 0 | 0 | 0 | 0 | 0 | 0 | 0 | 0 | 0 |
| 0 | 0 | 0 | 0 | 0 | 0 | 0 | 0 | 0 | 0 | 0 | 0 | 0 | 0 | 0 |
| 0 | 0 | 0 | 0 | 0 | 0 | 0 | 0 | 0 | 0 | 0 | 0 | 0 | 0 | 0 |
| 0 | 0 | 0 | 0 | 0 | 0 | 0 | 0 | 0 | 0 | 0 | 0 | 0 | 0 | 0 |
| 0 | 0 | 0 | 0 | 0 | 2 | 1 | 1 | 0 | 4 | 0 | 0 | 0 | 0 | 0 |
| 0 | 0 | 0 | 0 | 0 | 0 | 0 | 0 | 0 | 6 | 0 | 0 | 0 | 0 | 0 |
| 0 | 0 | 0 | 0 | 0 | 0 | 0 | 0 | 0 | 5 | 0 | 0 | 0 | 0 | 1 |
| 0 | 0 | 0 | 0 | 0 | 0 | 0 | 0 | 0 | 4 | 0 | 0 | 0 | 0 | 0 |
| 0 | 0 | 0 | 0 | 0 | 0 | 0 | 0 | 0 | 6 | 0 | 0 | 0 | 0 | 0 |
| 0 | 0 | 0 | 0 | 0 | 0 | 0 | 0 | 0 | 4 | 0 | 0 | 0 | 0 | 0 |
| 0 | 0 | 0 | 0 | 0 | 0 | 0 | 0 | 0 | 2 | 0 | 0 | 0 | 0 | 0 |
| 0 | 0 | 0 | 0 | 0 | 0 | 0 | 1 | 0 | 4 | 1 | 0 | 0 | 0 | 0 |
| 0 | 0 | 0 | 0 | 0 | 0 | 1 | 0 | 0 | 2 | 0 | 0 | 0 | 0 | 0 |
| 0 | 0 | 0 | 0 | 1 | 0 | 0 | 0 | 0 | 3 | 0 | 0 | 0 | 0 | 0 |
| 0 | 0 | 0 | 0 | 0 | 0 | 0 | 0 | 0 | 0 | 0 | 0 | 0 | 0 | 0 |
| 0 | 0 | 0 | 0 | 0 | 0 | 0 | 0 | 0 | 1 | 0 | 0 | 0 | 0 | 0 |
| 0 | 0 | 0 | 0 | 0 | 0 | 0 | 0 | 0 | 0 | 0 | 0 | 0 | 0 | 0 |
| 0 | 0 | 0 | 0 | 0 | 0 | 0 | 0 | 0 | 0 | 0 | 0 | 0 | 0 | 1 |
| 0 | 0 | 0 | 0 | 0 | 0 | 0 | 0 | 0 | 0 | 0 | 0 | 0 | 0 | 0 |
| 0 | 0 | 0 | 0 | 0 | 0 | 0 | 0 | 0 | 0 | 0 | 2 | 0 | 0 | 0 |
| 0 | 3 | 0 | 1 | 3 | 11 | 5 | 11 | 1 | 0 | 1 | 1 | 0 | 1 | 0 |
| 0 | 12 | 0 | 6 | 7 | 35 | 4 | 13 | 2 | 1 | 1 | 0 | 2 | 2 | 0 |
| 0 | 6 | 1 | 2 | 5 | 11 | 2 | 7 | 1 | 0 | 2 | 0 | 1 | 1 | 0 |

| PPTG_14894 | PPTG_14895 | PPTG_14896 | PPTG_14897 | PPTG_23634 | gene 757 | PPTG_14898 | PPTG_14899 | PPTG_23635 | gene 789 | gene 788 | PPTG_14900 | gene 787 | PPTG_14901 | PPTG_23636 | PPTG_23637 |
|---|---|---|---|---|---|---|---|---|---|---|---|---|---|---|---|
| 0 | 0 | 0 | 0 | 1539 | 0 | 0 | 0 | 0 | 0 | 0 | 0 | 0 | 0 | 0 | 0 |
| 0 | 0 | 0 | 0 | 1755 | 0 | 0 | 0 | 0 | 0 | 0 | 0 | 0 | 0 | 0 | 0 |
| 0 | 0 | 0 | 0 | 1514 | 0 | 0 | 0 | 0 | 0 | 0 | 0 | 0 | 0 | 0 | 0 |
| 0 | 0 | 0 | 0 | 1780 | 0 | 0 | 0 | 0 | 0 | 0 | 0 | 0 | 0 | 0 | 0 |
| 0 | 1 | 0 | 0 | 1297 | 0 | 0 | 0 | 0 | 0 | 0 | 0 | 0 | 0 | 2 | 0 |
| 0 | 0 | 0 | 0 | 1260 | 0 | 0 | 0 | 0 | 0 | 0 | 0 | 0 | 0 | 1 | 1 |
| 0 | 0 | 0 | 0 | 1119 | 0 | 0 | 0 | 0 | 0 | 0 | 0 | 0 | 0 | 0 | 1 |
| 0 | 0 | 0 | 0 | 1137 | 0 | 0 | 0 | 0 | 0 | 0 | 0 | 0 | 0 | 0 | 1 |
| 0 | 0 | 0 | 0 | 875 | 0 | 0 | 0 | 0 | 1 | 0 | 0 | 0 | 0 | 0 | 1 |
| 0 | 0 | 0 | 0 | 851 | 0 | 0 | 0 | 1 | 0 | 0 | 0 | 0 | 0 | 0 | 0 |
| 0 | 0 | 2 | 0 | 1016 | 0 | 0 | 0 | 0 | 0 | 0 | 0 | 0 | 0 | 0 | 0 |
| 0 | 0 | 1 | 0 | 752 | 0 | 0 | 0 | 0 | 0 | 0 | 0 | 0 | 0 | 0 | 0 |
| 0 | 1 | 0 | 0 | 917 | 0 | 0 | 0 | 0 | 0 | 0 | 0 | 0 | 0 | 0 | 0 |
| 0 | 1 | 0 | 0 | 601 | 0 | 0 | 0 | 0 | 0 | 0 | 0 | 0 | 0 | 1 | 0 |
| 0 | 0 | 0 | 0 | 594 | 0 | 0 | 0 | 1 | 0 | 0 | 0 | 0 | 0 | 0 | 0 |
| 0 | 1 | 0 | 1 | 857 | 0 | 0 | 0 | 0 | 0 | 0 | 0 | 0 | 0 | 0 | 0 |
| 0 | 2 | 0 | 0 | 861 | 0 | 0 | 0 | 0 | 0 | 0 | 0 | 0 | 1 | 0 | 0 |
| 0 | 0 | 0 | 0 | 796 | 0 | 0 | 0 | 0 | 0 | 0 | 0 | 0 | 0 | 0 | 0 |
| 0 | 1 | 0 | 0 | 1233 | 0 | 0 | 1 | 0 | 0 | 0 | 0 | 0 | 0 | 1 | 0 |
| 0 | 1 | 0 | 0 | 869 | 0 | 0 | 0 | 0 | 0 | 0 | 0 | 0 | 0 | 1 | 0 |
| 0 | 1 | 0 | 1 | 1621 | 0 | 0 | 0 | 0 | 0 | 0 | 0 | 0 | 0 | 1 | 0 |
| 0 | 0 | 0 | 0 | 1423 | 0 | 0 | 0 | 0 | 0 | 0 | 0 | 0 | 0 | 0 | 0 |
| 0 | 0 | 0 | 0 | 4125 | 0 | 0 | 0 | 0 | 0 | 0 | 0 | 0 | 0 | 0 | 0 |
| 0 | 0 | 0 | 0 | 1600 | 0 | 0 | 0 | 1 | 0 | 0 | 0 | 0 | 1 | 4 | 2 |
| 1 | 7 | 1 | 3 | 0 | 5 | 10 | 4 | 0 | 3 | 2 | 0 | 0 | 7 | 8 | 0 |
| 7 | 17 | 8 | 11 | 4 | 10 | 14 | 18 | 5 | 2 | 5 | 5 | 2 | 11 | 14 | 4 |
| 2 | 9 | 7 | 6 | 4 | 12 | 8 | 7 | 2 | 6 | 6 | 5 | 4 | 12 | 8 | 3 |

| PPTG_14903 | PPTG_14904 | PPTG_23638 | PPTG_14907 | PPTG_14909 | PPTG_23639 | PPTG_23640 | PPTG_14912 | PPTG_23641 | PPTG_23642 | PPTG_23643 | PPTG_14913 | PPTG_14917 | PPTG_23644 | PPTG_14918 |
|---|---|---|---|---|---|---|---|---|---|---|---|---|---|---|
| 23 | 0 | 2336 | 100 | 0 | 0 | 0 | 4823 | 0 | 0 | 0 | 0 | 4 | 0 | 0 |
| 29 | 0 | 3122 | 863 | 0 | 0 | 0 | ≥ 5000 | 0 | 0 | 0 | 0 | 115 | 0 | 0 |
| 30 | 0 | 2777 | 669 | 0 | 0 | 0 | ≥ 5000 | 0 | 0 | 0 | 0 | 0 | 0 | 0 |
| 29 | 0 | 3041 | 863 | 0 | 0 | 0 | ≥ 5000 | 0 | 0 | 0 | 0 | 115 | 0 | 0 |
| 4 | 0 | 1835 | 73 | 0 | 0 | 0 | 1749 | 0 | 0 | 0 | 0 | 0 | 0 | 0 |
| 13 | 0 | 1814 | 63 | 0 | 0 | 0 | 1853 | 0 | 0 | 0 | 0 | 0 | 0 | 0 |
| 7 | 0 | 1890 | 57 | 0 | 0 | 0 | 1575 | 0 | 0 | 0 | 0 | 0 | 0 | 0 |
| 7 | 0 | 1791 | 65 | 0 | 0 | 0 | 1707 | 0 | 0 | 0 | 0 | 0 | 0 | 0 |
| 26 | 0 | 2526 | 79 | 0 | 0 | 1 | 2537 | 0 | 0 | 0 | 2 | 0 | 0 | 0 |
| 50 | 0 | 2674 | 74 | 0 | 0 | 0 | 2185 | 0 | 0 | 0 | 0 | 0 | 0 | 0 |
| 32 | 0 | 2973 | 97 | 0 | 0 | 1 | 2709 | 0 | 0 | 0 | 2 | 0 | 0 | 0 |
| 38 | 0 | 2426 | 100 | 0 | 0 | 0 | 2617 | 0 | 0 | 0 | 1 | 0 | 0 | 0 |
| 32 | 0 | 2356 | 98 | 0 | 0 | 2 | 2402 | 0 | 0 | 0 | 0 | 0 | 0 | 0 |
| 33 | 0 | 1742 | 65 | 0 | 0 | 2 | 1912 | 0 | 0 | 0 | 0 | 0 | 0 | 0 |
| 38 | 0 | 1537 | 70 | 0 | 0 | 0 | 1873 | 0 | 0 | 0 | 0 | 0 | 0 | 1 |
| 55 | 0 | 1919 | 119 | 0 | 0 | 1 | 2324 | 0 | 0 | 0 | 1 | 1 | 0 | 0 |
| 44 | 0 | 2283 | 75 | 0 | 0 | 3 | 2341 | 0 | 0 | 0 | 0 | 0 | 0 | 0 |
| 28 | 0 | 1729 | 81 | 0 | 0 | 2 | 2201 | 0 | 1 | 0 | 0 | 0 | 0 | 0 |
| 7 | 0 | 1895 | 68 | 0 | 0 | 0 | 3192 | 0 | 0 | 0 | 0 | 0 | 0 | 0 |
| 18 | 0 | 1839 | 68 | 0 | 0 | 0 | 3154 | 0 | 1 | 0 | 0 | 0 | 0 | 0 |
| 6 | 1 | 1612 | 52 | 0 | 0 | 0 | 4893 | 0 | 1 | 0 | 0 | 0 | 0 | 0 |
| 5 | 0 | 2148 | 97 | 0 | 0 | 0 | 2968 | 0 | 0 | 0 | 0 | 183 | 0 | 0 |
| 4 | 0 | ≥ 5000 | 348 | 0 | 0 | 0 | ≥ 5000 | 0 | 0 | 0 | 0 | 293 | 0 | 0 |
| 11 | 0 | 3990 | 168 | 0 | 0 | 0 | 3518 | 0 | 0 | 0 | 0 | 145 | 0 | 0 |
| 0 | 6 | 9 | 0 | 0 | 4 | 4 | 20 | 0 | 0 | 0 | 8 | 2 | 3 | 1 |
| 1 | 15 | 16 | 3 | 3 | 8 | 0 | 55 | 0 | 0 | 0 | 13 | 5 | 2 | 2 |
| 0 | 15 | 16 | 1 | 4 | 2 | 0 | 21 | 1 | 0 | 1 | 9 | 1 | 2 | 1 |

| PPTG_14919 | PPTG_14920 | PPTG_14921 | PPTG_14922 | PPTG_14923 | PPTG_23645 | PPTG_14924 | PPTG_23646 | PPTG_14925 | PPTG_14926 | PPTG_14927 | PPTG_14928 | PPTG_14929 | PPTG_14931 | PPTG_23647 |
|---|---|---|---|---|---|---|---|---|---|---|---|---|---|---|
| 0 | 0 | 0 | 0 | 0 | 0 | 0 | 0 | 154 | 99 | 1994 | 113 | 127 | 564 | 17 |
| 0 | 1 | 0 | 0 | 0 | 0 | 0 | 0 | 193 | 92 | 2332 | 153 | 132 | 689 | 16 |
| 0 | 0 | 0 | 0 | 0 | 0 | 0 | 0 | 217 | 107 | 2165 | 138 | 117 | 539 | 11 |
| 0 | 0 | 0 | 0 | 0 | 0 | 0 | 1 | 212 | 129 | 2717 | 216 | 143 | 692 | 16 |
| 0 | 2 | 0 | 0 | 0 | 0 | 0 | 1 | 2239 | 17 | 601 | 66 | 28 | 689 | 84 |
| 0 | 1 | 0 | 0 | 0 | 0 | 0 | 0 | 2405 | 6 | 696 | 59 | 28 | 633 | 72 |
| 0 | 2 | 0 | 0 | 0 | 0 | 0 | 0 | 1869 | 19 | 539 | 65 | 20 | 736 | 74 |
| 0 | 2 | 0 | 0 | 0 | 0 | 0 | 0 | 2112 | 15 | 671 | 61 | 34 | 703 | 57 |
| 0 | 6 | 1 | 0 | 0 | 0 | 0 | 2 | 222 | 10 | 913 | 248 | 126 | 700 | 121 |
| 0 | 4 | 3 | 0 | 0 | 0 | 0 | 3 | 195 | 15 | 868 | 240 | 135 | 685 | 130 |
| 0 | 1 | 4 | 0 | 0 | 0 | 0 | 0 | 249 | 17 | 1031 | 241 | 119 | 876 | 164 |
| 0 | 0 | 1 | 0 | 0 | 0 | 0 | 6 | 296 | 13 | 997 | 319 | 179 | 1063 | 191 |
| 0 | 3 | 2 | 0 | 0 | 0 | 0 | 2 | 267 | 14 | 937 | 264 | 143 | 1065 | 180 |
| 0 | 0 | 2 | 0 | 0 | 0 | 0 | 1 | 205 | 13 | 761 | 226 | 148 | 573 | 126 |
| 0 | 0 | 1 | 0 | 0 | 0 | 0 | 0 | 184 | 10 | 678 | 227 | 131 | 505 | 118 |
| 0 | 0 | 0 | 0 | 0 | 0 | 0 | 0 | 312 | 10 | 872 | 310 | 190 | 697 | 163 |
| 0 | 4 | 0 | 0 | 0 | 0 | 0 | 0 | 239 | 5 | 873 | 286 | 171 | 1072 | 158 |
| 0 | 1 | 0 | 0 | 0 | 0 | 0 | 2 | 258 | 19 | 845 | 272 | 153 | 767 | 169 |
| 0 | 2 | 0 | 0 | 0 | 0 | 0 | 2 | 426 | 18 | 1252 | 148 | 39 | 695 | 75 |
| 0 | 2 | 1 | 0 | 0 | 0 | 0 | 1 | 307 | 44 | 1237 | 279 | 86 | 837 | 84 |
| 0 | 2 | 1 | 0 | 0 | 0 | 0 | 1 | 536 | 43 | 1949 | 278 | 105 | 983 | 130 |
| 0 | 0 | 0 | 0 | 0 | 0 | 0 | 0 | 1627 | 35 | 841 | 409 | 68 | 1835 | 320 |
| 0 | 0 | 0 | 0 | 0 | 0 | 0 | 0 | 3798 | 108 | 2847 | 837 | 152 | ≥ 5000 | 860 |
| 0 | 0 | 0 | 0 | 0 | 0 | 0 | 0 | 2407 | 80 | 1223 | 573 | 142 | 2180 | 416 |
| 4 | 14 | 16 | 1 | 0 | 0 | 2 | 10 | 4 | 0 | 8 | 1 | 0 | 5 | 0 |
| 4 | 25 | 35 | 1 | 3 | 0 | 3 | 26 | 10 | 1 | 10 | 2 | 0 | 19 | 1 |
| 3 | 21 | 28 | 0 | 1 | 0 | 2 | 13 | 9 | 1 | 5 | 3 | 0 | 4 | 1 |

| PPTG_14932 | PPTG_14933 | PPTG_14934 | PPTG_23648 | PPTG_23649 | PPTG_23650 | PPTG_23651 | PPTG_23652 | PPTG_14935 | PPTG_14936 | PPTG_14937 | PPTG_23653 | PPTG_14938 | PPTG_23654 | PPTG_23655 |
|---|---|---|---|---|---|---|---|---|---|---|---|---|---|---|
| 6 | 6 | 0 | 0 | 0 | 1 | 118 | 67 | 0 | 1778 | 64 | 0 | 0 | 1 | 0 |
| 12 | 10 | 0 | 0 | 0 | 1 | 128 | 84 | 0 | 2406 | 59 | 0 | 0 | 0 | 0 |
| 7 | 8 | 0 | 0 | 0 | 0 | 136 | 61 | 1 | 2164 | 64 | 0 | 0 | 0 | 0 |
| 6 | 12 | 0 | 0 | 0 | 4 | 153 | 105 | 0 | 2281 | 53 | 0 | 0 | 0 | 0 |
| 72 | 7 | 0 | 0 | 0 | 2 | 79 | 77 | 1 | 1228 | 133 | 0 | 0 | 0 | 0 |
| 73 | 5 | 0 | 0 | 0 | 4 | 99 | 64 | 1 | 1210 | 145 | 0 | 0 | 0 | 0 |
| 75 | 11 | 0 | 0 | 0 | 2 | 108 | 92 | 1 | 1202 | 152 | 0 | 0 | 0 | 0 |
| 66 | 3 | 0 | 0 | 0 | 3 | 78 | 90 | 0 | 1194 | 172 | 0 | 0 | 2 | 0 |
| 201 | 52 | 0 | 2 | 0 | 8 | 56 | 57 | 2 | 2094 | 271 | 2 | 0 | 2 | 0 |
| 177 | 72 | 2 | 0 | 0 | 10 | 46 | 36 | 0 | 2221 | 243 | 0 | 0 | 0 | 0 |
| 218 | 63 | 0 | 0 | 0 | 12 | 47 | 32 | 2 | 2495 | 319 | 0 | 0 | 0 | 0 |
| 322 | 73 | 0 | 0 | 0 | 16 | 36 | 28 | 1 | 1963 | 304 | 0 | 0 | 0 | 0 |
| 359 | 57 | 0 | 1 | 0 | 9 | 48 | 34 | 1 | 1820 | 311 | 0 | 0 | 0 | 0 |
| 208 | 46 | 1 | 1 | 0 | 5 | 33 | 29 | 1 | 1426 | 232 | 0 | 0 | 0 | 0 |
| 159 | 44 | 1 | 3 | 0 | 8 | 36 | 21 | 1 | 1202 | 280 | 0 | 0 | 0 | 0 |
| 261 | 48 | 0 | 0 | 0 | 17 | 36 | 24 | 4 | 1456 | 320 | 0 | 0 | 0 | 0 |
| 354 | 49 | 0 | 1 | 0 | 13 | 61 | 40 | 0 | 1743 | 319 | 0 | 0 | 0 | 0 |
| 311 | 31 | 0 | 0 | 0 | 3 | 37 | 31 | 1 | 1313 | 328 | 0 | 0 | 0 | 0 |
| 131 | 34 | 0 | 0 | 0 | 5 | 51 | 48 | 0 | 1435 | 216 | 0 | 0 | 0 | 0 |
| 147 | 44 | 0 | 0 | 0 | 2 | 49 | 31 | 0 | 1364 | 275 | 0 | 0 | 0 | 0 |
| 153 | 44 | 0 | 0 | 0 | 7 | 85 | 71 | 0 | 976 | 382 | 0 | 0 | 0 | 0 |
| 158 | 57 | 0 | 0 | 0 | 1 | 19 | 7 | 0 | 1480 | 58 | 0 | 0 | 0 | 0 |
| 408 | 172 | 0 | 0 | 0 | 3 | 30 | 15 | 0 | ≥ 5000 | 295 | 0 | 0 | 0 | 0 |
| 110 | 62 | 0 | 0 | 0 | 1 | 24 | 16 | 0 | 3102 | 103 | 0 | 0 | 2 | 0 |
| 1 | 2 | 1 | 9 | 1 | 4 | 0 | 0 | 14 | 7 | 1 | 1 | 5 | 14 | 0 |
| 5 | 2 | 2 | 16 | 1 | 11 | 0 | 2 | 28 | 12 | 1 | 2 | 8 | 33 | 3 |
| 1 | 2 | 5 | 9 | 0 | 7 | 1 | 0 | 25 | 12 | 1 | 3 | 12 | 38 | 2 |

| PPTG_14941 | PPTG_14942 | PPTG_14943 | PPTG_14944 | PPTG_14945 | gene403 | gene404 | PPTG_23656 | gene407 | PPTG_14946 | PPTG_14947 | PPTG_23657 | PPTG_14950 | PPTG_14951 | PPTG_23658 | PPTG_23659 |
|---|---|---|---|---|---|---|---|---|---|---|---|---|---|---|---|
| 0 | 16 | 8 | 5 | 205 | 0 | 0 | 0 | 0 | 103 | 0 | 0 | 0 | 0 | 412 | 5 |
| 0 | 18 | 3 | 7 | 138 | 0 | 0 | 0 | 0 | 111 | 0 | 0 | 0 | 0 | 546 | 15 |
| 0 | 36 | 10 | 9 | 267 | 0 | 0 | 0 | 0 | 119 | 0 | 0 | 0 | 0 | 447 | 12 |
| 0 | 31 | 5 | 8 | 214 | 0 | 0 | 0 | 0 | 137 | 0 | 0 | 0 | 1 | 557 | 13 |
| 0 | 52 | 17 | 51 | 668 | 0 | 0 | 0 | 0 | 21 | 0 | 0 | 0 | 0 | 548 | 65 |
| 0 | 50 | 7 | 35 | 623 | 0 | 0 | 0 | 0 | 19 | 0 | 0 | 0 | 0 | 519 | 61 |
| 0 | 42 | 16 | 30 | 619 | 0 | 0 | 0 | 0 | 25 | 0 | 0 | 0 | 0 | 579 | 37 |
| 1 | 39 | 13 | 35 | 542 | 0 | 0 | 2 | 0 | 20 | 0 | 0 | 0 | 0 | 554 | 39 |
| 0 | 40 | 24 | 24 | 637 | 1 | 0 | 2 | 0 | 96 | 0 | 1 | 0 | 0 | 599 | 31 |
| 0 | 42 | 25 | 30 | 697 | 0 | 0 | 0 | 0 | 123 | 0 | 0 | 0 | 2 | 584 | 34 |
| 0 | 47 | 17 | 47 | 573 | 2 | 2 | 0 | 0 | 122 | 1 | 0 | 0 | 0 | 625 | 22 |
| 0 | 31 | 36 | 34 | 670 | 5 | 11 | 1 | 1 | 145 | 0 | 0 | 0 | 0 | 717 | 22 |
| 0 | 52 | 18 | 29 | 669 | 1 | 2 | 0 | 1 | 93 | 0 | 0 | 0 | 1 | 676 | 28 |
| 0 | 28 | 32 | 19 | 569 | 1 | 4 | 0 | 1 | 102 | 0 | 0 | 0 | 1 | 441 | 31 |
| 0 | 34 | 25 | 30 | 1142 | 1 | 1 | 0 | 0 | 109 | 0 | 0 | 0 | 0 | 423 | 26 |
| 0 | 42 | 43 | 44 | 1203 | 1 | 6 | 0 | 1 | 136 | 0 | 0 | 0 | 1 | 562 | 28 |
| 0 | 28 | 37 | 30 | 1080 | 2 | 1 | 0 | 0 | 103 | 0 | 0 | 0 | 1 | 672 | 28 |
| 0 | 36 | 37 | 34 | 1160 | 1 | 1 | 1 | 1 | 108 | 0 | 0 | 0 | 2 | 557 | 36 |
| 0 | 62 | 22 | 21 | 456 | 2 | 1 | 0 | 0 | 25 | 0 | 0 | 0 | 0 | 466 | 38 |
| 0 | 62 | 14 | 22 | 867 | 2 | 2 | 1 | 0 | 44 | 0 | 0 | 0 | 0 | 520 | 25 |
| 0 | 86 | 7 | 18 | 1290 | 2 | 1 | 0 | 0 | 33 | 0 | 0 | 0 | 0 | 652 | 55 |
| 0 | 44 | 20 | 12 | 100 | 0 | 0 | 0 | 0 | 32 | 0 | 0 | 0 | 0 | 674 | 0 |
| 0 | 255 | 59 | 17 | 391 | 0 | 0 | 0 | 0 | 42 | 0 | 0 | 0 | 0 | 1859 | 0 |
| 1 | 70 | 64 | 10 | 240 | 0 | 0 | 0 | 0 | 36 | 0 | 0 | 0 | 0 | 836 | 0 |
| 0 | 0 | 1 | 0 | 6 | 0 | 1 | 0 | 1 | 1 | 0 | 0 | 0 | 0 | 4 | 0 |
| 0 | 0 | 2 | 0 | 6 | 0 | 1 | 2 | 1 | 2 | 1 | 0 | 2 | 0 | 7 | 0 |
| 3 | 2 | 1 | 1 | 8 | 0 | 0 | 2 | 2 | 4 | 0 | 1 | 1 | 0 | 3 | 0 |

| PPTG_14954 | PPTG_14955 | PPTG_14956 | PPTG_14957 | PPTG_23660 | PPTG_23661 | PPTG_14958 | PPTG_14959 | PPTG_14960 | PPTG_23662 | gene 757 |
|---|---|---|---|---|---|---|---|---|---|---|
| 0 | 0 | 512 | 0 | 0 | 0 | 0 | 0 | 3 | 0 | 0 |
| 0 | 0 | 560 | 0 | 0 | 0 | 1 | 0 | 2 | 0 | 0 |
| 0 | 0 | 529 | 0 | 0 | 0 | 0 | 0 | 1 | 0 | 0 |
| 0 | 0 | 612 | 0 | 0 | 0 | 0 | 0 | 2 | 0 | 0 |
| 0 | 0 | 485 | 0 | 0 | 0 | 0 | 0 | 0 | 0 | 0 |
| 0 | 0 | 492 | 0 | 0 | 0 | 1 | 0 | 1 | 0 | 0 |
| 0 | 0 | 533 | 0 | 0 | 0 | 0 | 0 | 2 | 0 | 0 |
| 0 | 0 | 493 | 0 | 0 | 0 | 1 | 0 | 1 | 0 | 0 |
| 0 | 0 | 557 | 0 | 0 | 0 | 6 | 0 | 0 | 0 | 0 |
| 0 | 0 | 594 | 0 | 0 | 0 | 2 | 0 | 1 | 0 | 0 |
| 0 | 0 | 557 | 0 | 0 | 0 | 3 | 0 | 0 | 0 | 0 |
| 0 | 0 | 491 | 0 | 0 | 0 | 8 | 0 | 0 | 0 | 0 |
| 0 | 0 | 620 | 0 | 0 | 0 | 7 | 0 | 0 | 1 | 0 |
| 0 | 0 | 435 | 0 | 0 | 0 | 6 | 0 | 0 | 1 | 0 |
| 0 | 0 | 422 | 0 | 0 | 0 | 2 | 0 | 0 | 0 | 0 |
| 0 | 0 | 578 | 0 | 0 | 0 | 1 | 1 | 0 | 0 | 0 |
| 0 | 0 | 617 | 0 | 0 | 0 | 1 | 0 | 0 | 0 | 0 |
| 0 | 0 | 517 | 7 | 0 | 0 | 1 | 0 | 0 | 0 | 0 |
| 0 | 0 | 503 | 0 | 0 | 0 | 2 | 0 | 0 | 0 | 0 |
| 0 | 0 | 406 | 0 | 0 | 0 | 10 | 0 | 0 | 0 | 0 |
| 0 | 0 | 480 | 0 | 0 | 0 | 4 | 1 | 0 | 0 | 0 |
| 0 | 0 | 217 | 0 | 0 | 0 | 0 | 0 | 0 | 0 | 0 |
| 0 | 0 | 505 | 0 | 0 | 0 | 2 | 0 | 0 | 0 | 0 |
| 0 | 0 | 311 | 0 | 0 | 0 | 0 | 0 | 0 | 0 | 0 |
| 1 | 0 | 0 | 0 | 2 | 1 | 0 | 1 | 2 | 2 | 5 |
| 0 | 1 | 1 | 1 | 0 | 0 | 2 | 11 | 2 | 8 | 10 |
| 1 | 2 | 3 | 2 | 3 | 0 | 1 | 5 | 2 | 20 | 12 |
